# Supplementary material for: Do cash transfers alleviate common mental disorders in low- and middle-income countries? A systematic review and meta-analysis
Source: PLoS One. 2023 Feb 22;18(2):e0281283. doi: 10.1371/journal.pone.0281283 (PMC9946251; doi:10.1371/journal.pone.0281283)
Supplement: S1 File — (DOCX) [file pone.0281283.s001.docx]

**Do cash transfers alleviate common mental disorders in low- and middle-income countries? A systematic review and meta-analysis**

***Supplementary Appendices***

**Supplementary Appendix S1. Example Search Syntaxes**

**SCOPUS (searched April 15, 2020)**

| 1 | Cash transfer programs | TITLE-ABS-KEY("cash transfer*" OR "social transfer*" OR "income transfer*" OR "income grant*" OR "income support" OR "social grant*" OR "basic grant*" OR "basic income" OR "minimum income" OR "social assistance" OR "social security" OR "social welfare" OR "social pension*" OR "non-contributory pension*" OR "old age pension*" OR "old age grant*" OR "old age benefit*" OR "child grant*" OR "child benefit*")  *Sources: (Baird et al., 2013b), (Bastagli et al., 2016)* |
| --- | --- | --- |
| 2 | Mental health | TITLE-ABS-KEY("mental disorder*" OR "mental health" OR "mental illness*" OR "mental ill health" OR "common mental disorder*" OR "stress disorder*" OR "stress" OR "psychosocial stress" OR "anxiety" OR "anxiety disorder*" OR "depression" OR "depressive disorder*" OR "negative affective" OR "affective disorder*" OR "wellbeing" OR "well-being" OR ((psychological OR psychosocial OR emotional) W/ (wellbeing OR well-being))) |
| 3 | LMICs by name | TITLE-ABS-KEY( afghanistan OR albania OR algeria OR angola OR antigua OR barbuda OR argentina OR armenia OR armenian OR aruba OR azerbaijan OR bahrain OR bangladesh OR barbados OR benin OR byelarus OR byelorussian OR belarus OR belorussian OR belorussia OR belize OR bhutan OR bolivia OR bosnia OR herzegovina OR hercegovina OR botswana OR brasil OR brazil OR bulgaria OR "Burkina Faso" OR "Burkina Fasso" OR "Upper Volta" OR burundi OR urundi OR cambodia OR "Khmer Republic" OR kampuchea OR cameroon OR cameroons OR cameron OR camerons OR "Cape Verde" OR "Central African Republic" OR chad OR chile OR china OR colombia OR comoros OR "Comoro Islands" OR comores OR mayotte OR congo OR zaire OR "Costa Rica" OR "Cote d'Ivoire" OR "Ivory Coast" OR croatia OR cuba OR cyprus OR czechoslovakia OR "Czech Republic" OR slovakia OR "Slovak Republic" OR djibouti OR "French Somaliland" OR dominica OR "Dominican Republic" OR "East Timor" OR "East Timur" OR "Timor Leste" OR ecuador OR egypt OR "United Arab Republic" OR "El Salvador" OR eritrea OR estonia OR ethiopia OR fiji OR gabon OR "Gabonese Republic" OR gambia OR gaza OR "Georgia Republic" OR "Georgian Republic" OR ghana OR "Gold Coast" OR greece OR grenada OR guatemala OR guinea OR guam OR guiana OR guyana OR haiti OR honduras OR hungary OR india OR maldives OR indonesia OR iran OR iraq OR "Isle of Man" OR jamaica OR jordan OR kazakhstan OR kazakh OR kenya OR kiribati OR korea OR kosovo OR kyrgyzstan OR kirghizia OR "Kyrgyz Republic" OR kirghiz OR kirgizstan OR "Lao PDR" OR laos OR latvia OR lebanon OR lesotho OR basutoland OR liberia OR libya OR lithuania OR macedonia OR madagascar OR "Malagasy Republic" OR malaysia OR malaya OR malay OR sabah OR sarawak OR malawi OR nyasaland OR mali OR malta OR "Marshall Islands" OR mauritania OR mauritius OR "Agalega Islands" OR mexico OR micronesia OR "Middle East" OR moldova OR moldovia OR moldovian OR mongolia OR montenegro OR morocco OR ifni OR mozambique OR myanmar OR myanma OR burma OR namibia OR nepal OR "Netherlands Antilles" OR "New Caledonia" OR nicaragua OR niger OR nigeria OR "Northern Mariana Islands" OR oman OR muscat OR pakistan OR palau OR palestine OR panama OR paraguay OR peru OR philippines OR philipines OR phillipines OR phillippines OR poland OR portugal OR "Puerto Rico" OR romania OR rumania OR roumania OR russia OR russian OR rwanda OR ruanda OR "Saint Kitts" OR "St Kitts" OR nevis OR "Saint Lucia" OR "St Lucia" OR "Saint Vincent" OR "St Vincent" OR grenadines OR samoa OR "Samoan Islands" OR "Navigator Island" OR "Navigator Islands" OR "Sao Tome" OR "Saudi Arabia" OR senegal OR serbia OR montenegro OR seychelles OR "Sierra Leone" OR slovenia OR "Sri Lanka" OR ceylon OR "Solomon Islands" OR somalia OR "South Africa" OR sudan OR suriname OR surinam OR swaziland OR syria OR tajikistan OR tadzhikistan OR tadjikistan OR tadzhik OR tanzania OR thailand OR togo OR "Togolese Republic" OR tonga OR trinidad OR tobago OR tunisia OR turkey OR turkmenistan OR turkmen OR uganda OR ukraine OR uruguay OR ussr OR "Soviet Union" OR "Union of Soviet Socialist Republics" OR uzbekistan OR uzbek OR vanuatu OR "New Hebrides" OR venezuela OR vietnam OR "Viet Nam" OR “west bank” OR yemen OR yugoslavia OR zambia OR zimbabwe OR rhodesia)  Source: (EPOC, 2012) |
| 4 | LMICs by region | TITLE-ABS-KEY ( africa  OR  asia  OR  caribbean  OR  "West Indies"  OR  "South America"  OR  "Latin America"  OR  "Central America" )  *Source: (EPOC, 2012)* |
| 5 | LMICs overarching | TITLE-ABS-KEY ( ( ( developing OR "less* developed" OR "under developed" OR underdeveloped OR "middle income" OR "low* income" OR underserved OR "under served" OR deprived OR poor* ) W/ ( countr* OR nation* OR population* OR world ) ) OR ( ( developing OR "less* developed" OR "under developed" OR underdeveloped OR "middle income" OR "low* income" ) W/ ( economy OR economies ) ) OR ( low* W/ ( gdp OR gnp OR "gross domestic" OR "gross national" ) ) OR ( low W/3 middle W/3 countr* ) OR ( lmic OR lmics OR "third world" OR "lami countr*" ) OR "transitional countr*" )  *Source: (EPOC, 2012)* |
| 6 | 3 OR 4 OR 5 | |
| 7 | 1 AND 2 AND 6 | |

**PsycInfo** **(searched April 16, 2020)**

| 1 | ("cash transfer$" or "social transfer$" or "income transfer$" or "income grant$" or "income support" or "social grant$" or "basic grant$" or "basic income" or "minimum income" or "social assistance" or "social security" or "social welfare" or "social pension$" or "non-contributory pension$" or "old age pension$" or "old age grant$" or "old age benefit$" or "child grant$" or "child benefit$").ab,hw,id,mh,ti.  *Sources:* (Baird et al., 2013b)*, (Bastagli et al., 2016)* |
| --- | --- |
| 2 | ("mental disorder$" or "mental health" or "mental illness*" or "mental ill health" or "common mental disorder$" or "stress disorder$" or "stress" or "psychosocial stress" or "anxiety" or "anxiety disorder$" or "depression" or "depressive disorder$" or "negative affective" or "affective disorder$" or "wellbeing" or "well-being" or ((psychological or psychosocial or emotional) adj (wellbeing or well-being))).ab,hw,id,mh,ti.  *Sources: (Baird et al., 2013b), (Bastagli et al., 2016)* |
| 3 | (Afghanistan or Albania or Algeria or Angola or Antigua or Barbuda or Argentina or Armenia or Armenian or Aruba or Azerbaijan or Bahrain or Bangladesh or Barbados or Benin or Byelarus or Byelorussian or Belarus or Belorussian or Belorussia or Belize or Bhutan or Bolivia or Bosnia or Herzegovina or Hercegovina or Botswana or Brasil or Brazil or Bulgaria or "Burkina Faso" or "Burkina Fasso" or "Upper Volta" or Burundi or Urundi or Cambodia or "Khmer Republic" or Kampuchea or Cameroon or Cameroons or Cameron or Camerons or "Cape Verde" or "Central African Republic" or Chad or Chile or China or Colombia or Comoros or "Comoro Islands" or Comores or Mayotte or Congo or Zaire or "Costa Rica" or "Cote d'Ivoire" or "Ivory Coast" or Croatia or Cuba or Cyprus or Czechoslovakia or "Czech Republic" or Slovakia or "Slovak Republic" or Djibouti or "French Somaliland" or Dominica or "Dominican Republic" or "East Timor" or "East Timur" or "Timor Leste" or Ecuador or Egypt or "United Arab Republic" or "El Salvador" or Eritrea or Estonia or Ethiopia or Fiji or Gabon or "Gabonese Republic" or Gambia or Gaza or "Georgia Republic" or "Georgian Republic" or Ghana or "Gold Coast" or Greece or Grenada or Guatemala or Guinea or Guam or Guiana or Guyana or Haiti or Honduras or Hungary or India or Maldives or Indonesia or Iran or Iraq or "Isle of Man" or Jamaica or Jordan or Kazakhstan or Kazakh or Kenya or Kiribati or Korea or Kosovo or Kyrgyzstan or Kirghizia or "Kyrgyz Republic" or Kirghiz or Kirgizstan or "Lao PDR" or Laos or Latvia or Lebanon or Lesotho or Basutoland or Liberia or Libya or Lithuania or Macedonia or Madagascar or "Malagasy Republic" or Malaysia or Malaya or Malay or Sabah or Sarawak or Malawi or Nyasaland or Mali or Malta or "Marshall Islands" or Mauritania or Mauritius or "Agalega Islands" or Mexico or Micronesia or "Middle East" or Moldova or Moldovia or Moldovian or Mongolia or Montenegro or Morocco or Ifni or Mozambique or Myanmar or Myanma or Burma or Namibia or Nepal or "Netherlands Antilles" or "New Caledonia" or Nicaragua or Niger or Nigeria or "Northern Mariana Islands" or Oman or Muscat or Pakistan or Palau or Palestine or Panama or Paraguay or Peru or Philippines or Philipines or Phillipines or Phillippines or Poland or Portugal or "Puerto Rico" or Romania or Rumania or Roumania or Russia or Russian or Rwanda or Ruanda or "Saint Kitts" or "St Kitts" or Nevis or "Saint Lucia" or "St Lucia" or "Saint Vincent" or "St Vincent" or Grenadines or Samoa or "Samoan Islands" or "Navigator Island" or "Navigator Islands" or "Sao Tome" or "Saudi Arabia" or Senegal or Serbia or Montenegro or Seychelles or "Sierra Leone" or Slovenia or "Sri Lanka" or Ceylon or "Solomon Islands" or Somalia or "South Africa" or Sudan or Suriname or Surinam or Swaziland or Syria or Tajikistan or Tadzhikistan or Tadjikistan or Tadzhik or Tanzania or Thailand or Togo or "Togolese Republic" or Tonga or Trinidad or Tobago or Tunisia or Turkey or Turkmenistan or Turkmen or Uganda or Ukraine or Uruguay or USSR or "Soviet Union" or "Union of Soviet Socialist Republics" or Uzbekistan or Uzbek or Vanuatu or "New Hebrides" or Venezuela or Vietnam or "Viet Nam" or “West Bank” or Yemen or Yugoslavia or Zambia or Zimbabwe or Rhodesia or (Africa or Asia or Caribbean or "West Indies" or "South America" or "Latin America" or "Central America") or (((developing or "less* developed" or "under developed" or underdeveloped or "middle income" or "low* income" or underserved or "under served" or deprived or poor*) adj (countr* or nation? or population? or world)) or ((developing or "less* developed" or "under developed" or underdeveloped or "middle income" or "low* income") adj (economy or economies)) or (low* adj (gdp or gnp or "gross domestic" or "gross national")) or (low adj3 middle adj3 countr*) or (lmic or lmics or "third world" or "lami countr*") or "transitional countr*")).ab,hw,ti.  *Source: (EPOC, 2012)* |
| 4 | 1 AND 2 AND 3 |

**Embase (searched April 17, 2020)**

| 1 | ("cash transfer$" or "social transfer$" or "income transfer$" or "income grant$" or "income support" or "social grant$" or "basic grant$" or "basic income" or "minimum income" or "social assistance" or "social security" or "social welfare" or "social pension$" or "non-contributory pension$" or "old age pension$" or "old age grant$" or "old age benefit$" or "child grant$" or "child benefit$").ab,hw,kw,ot,ti. |
| --- | --- |
| 2 | ("mental disorder$" or "mental health" or "mental illness*" or "mental ill health" or "common mental disorder$" or "stress disorder$" or "stress" or "psychosocial stress" or "anxiety" or "anxiety disorder$" or "depression" or "depressive disorder$" or "negative affective" or "affective disorder$" or "wellbeing" or "well-being" or ((psychological or psychosocial or emotional) adj (wellbeing or well-being))).ab,hw,kw,ot,ti. |
| 3 | (Afghanistan or Albania or Algeria or Angola or Antigua or Barbuda or Argentina or Armenia or Armenian or Aruba or Azerbaijan or Bahrain or Bangladesh or Barbados or Benin or Byelarus or Byelorussian or Belarus or Belorussian or Belorussia or Belize or Bhutan or Bolivia or Bosnia or Herzegovina or Hercegovina or Botswana or Brasil or Brazil or Bulgaria or "Burkina Faso" or "Burkina Fasso" or "Upper Volta" or Burundi or Urundi or Cambodia or "Khmer Republic" or Kampuchea or Cameroon or Cameroons or Cameron or Camerons or "Cape Verde" or "Central African Republic" or Chad or Chile or China or Colombia or Comoros or "Comoro Islands" or Comores or Mayotte or Congo or Zaire or "Costa Rica" or "Cote d'Ivoire" or "Ivory Coast" or Cr! oatia or Cuba or Cyprus or Czechoslovakia or "Czech Republic" or Slovakia or "Slovak Republic" or Djibouti or "French Somaliland" or Dominica or "Dominican Republic" or "East Timor" or "East Timur" or "Timor Leste" or Ecuador or Egypt or "United Arab Republic" or "El Salvador" or Eritrea or Estonia or Ethiopia or Fiji or Gabon or "Gabonese Republic" or Gambia or Gaza or "Georgia Republic" or "Georgian Republic" or Ghana or "Gold Coast" or Greece or Grenada or Guatemala or Guinea or Guam or Guiana or Guyana or Haiti or Honduras or Hungary or India or Maldives or Indonesia or Iran or Iraq or "Isle of Man" or Jamaica or Jordan or Kazakhstan or Kazakh or Kenya or Kiribati or Korea or Kosovo or Kyrgyzstan or Kirghizia or "Kyrgyz Republic" or Kirghiz or Kirgizstan or "Lao PDR" or Laos or Latvia or Lebanon or Lesotho or Basutoland or Liberia or Libya or Lithuania or Macedonia or Madagascar or "Malagasy Republic" or Malaysia or Malaya or Malay or Sabah or Sarawak or Malawi or Nyasaland or Mali or Malta or "Marshall Islands" or Mauritania or Mauritius or "Agalega Islands" or Mexico or Micronesia or "Middle East" or Moldova or Moldovia or Moldovian or Mongolia or Montenegro or Morocco or Ifni or Mozambique or Myanmar or Myanma or Burma or Namibia or Nepal or "Netherlands Antilles" or "New Caledonia" or Nicaragua or Niger or Nigeria or "Northern Mariana Islands" or Oman or Muscat or Pakistan or Palau or Palestine or Panama or Paraguay or Peru or Philippines or Philipines or Phillipines or Phillippines or Poland or Portugal or "Puerto Rico" or Romania or Rumania or Roumania or Russia or Russian or Rwanda or Ruanda or "Saint Kitts" or "St Kitts" or Nevis or "Saint Lucia" or "St Lucia" or "Saint Vincent" or "St Vincent" or Grenadines or Samoa or "Samoan Islands" or "Navigator Island" or "Navigator Islands" or "Sao Tome" or "Saudi Arabia" or Senegal or Serbia or Montenegro or Seychelles or "Sierra Leone" or Slovenia or "Sri Lanka" or Ceylon or "Solomon Islands" or Somalia or "South Africa" or Sudan or Suriname or Suri! nam or Sw! aziland or Syria or Tajikistan or Tadzhikistan or Tadjikistan or Tadzhik or Tanzania or Thailand or Togo or "Togolese Republic" or Tonga or Trinidad or Tobago or Tunisia or Turkey or Turkmenistan or Turkmen or Uganda or Ukraine or Uruguay or USSR or "Soviet Union" or "Union of Soviet Socialist Republics" or Uzbekistan or Uzbek or Vanuatu or "New Hebrides" or Venezuela or Vietnam or "Viet Nam" or West Bank or Yemen or Yugoslavia or Zambia or Zimbabwe or Rhodesia).ab,hw,kw,ot,ti.  *Source: (EPOC, 2012)* |
| 4 | (Africa or Asia or Caribbean or "West Indies" or "South America" or "Latin America" or "Central America").ab,hw,kw,ot,ti.  *Source: (EPOC, 2012)* |
| 5 | (((developing or "less* developed" or "under developed" or underdeveloped or "middle income" or "low* income" or underserved or "under served" or deprived or poor*) adj (countr* or nation? or population? or world)) or ((developing or "less* developed" or "under developed" or underdeveloped or "middle income" or "low* income") adj (economy or economies)) or (low* adj (gdp or gnp or "gross domestic" or "gross national")) or (low adj3 middle adj3 countr*) or (lmic or lmics or "third world" or "lami countr*") or "transitional countr*").ab,hw,kw,ot,ti.  *Source: (EPOC, 2012)* |
| 6 | 3 or 4 or 5 |
| 7 | 1 and 2 and 6 |

**3ie Evidence Database (searched April 17, 2020)**

("Cash transfer" OR "social transfer" OR "income transfer" OR "income support" OR "income grant" OR "social grant" OR "basic grant" OR "Minimum income" OR "social assistance" OR "social pension" OR or "non-contributory pension" OR "old age pension" OR "old age grant" OR "old age benefit" OR"child grant" OR "child benefit") AND ("mental disorder" OR "mental health" OR "mental illness" OR "well-being" OR "wellbeing" OR "depression" OR "stress" OR "anxiety" OR "depressive disorder")

**Supplementary Appendix S2.** **List of excluded studies**

| **Reason for exclusion** | **Paper** |
| --- | --- |
| Wrong intervention (e.g. cash-plus, in-kind transfers, microloans) | (Amin et al., 1998) |
|  | (Andrew et al., 2018) |
|  | (Banerjee et al., 2011) |
|  | (Banerjee et al., 2017) |
|  | (Banerjee et al., 2018) |
|  | (Bhanot et al., 2018) |
|  | (Blattman and Annan, 2011) |
|  | (Blattman and Annan, 2015) |
|  | (Blattman et al., 2016) |
|  | (Burmaster et al., 2015) |
|  | (Green et al., 2016) |
|  | (Karimli et al., 2019) |
|  | (Macours et al., 2012) |
|  | (Özler et al., 2020) |
|  | (Rocha et al., 2011) |
|  | (Tsaneva and Balakrishnan, 2019) |
|  | (Undurraga et al., 2016) |
| Wrong outcome (e.g. health/poverty/educational indicators, subjective wellbeing) | (Aker, 2017) |
|  | (Akresh et al., 2012) |
|  | (Alcazar et al., 2016) |
|  | (Almås et al., 2018) |
|  | (Amarante et al., 2016) |
|  | (Attah et al., 2016) |
|  | (Attanasio and Mesnard, 2006) |
|  | (Bagolin, 2017) |
|  | (Baird et al., 2013a) |
|  | (Baird et al., 2011) |
|  | (Barrera-Osorio et al., 2008) |
|  | (Barrera-Osorio et al., 2017) |
|  | (Barrientos and Villa, 2015) |
|  | (Behrman and Parker, 2010) |
|  | (Behrman and Parker, 2011) |
|  | (Behrman et al., 2012) |
|  | (Behrman et al., 2009) |
|  | (Benedetti et al., 2016) |
|  | (Berniell et al., 2014) |
|  | (Blattman et al., 2014) |
|  | (Blattman et al., 2014) |
|  | (Bobonis, 2011) |
|  | (Bobonis et al., 2013) |
|  | (Brenes-Camacho, 2011) |
|  | (Buller et al., 2016) |
|  | (Cahyadi et al., 2018) |
|  | (Christian et al., 2019) |
|  | (Coetzee, 2013) |
|  | (Crea et al., 2015) |
|  | (Cunha et al., 2019) |
|  | (D’Aoust et al., 2013) |
|  | (Dake et al., 2018) |
|  | (de Oliveira et al., 2017) |
|  | (Delgado et al., 2018) |
|  | (Ding, 2017) |
|  | (Edmonds and Schady, 2012) |
|  | (Eremina et al., 2016) |
|  | (Evans et al., 2014) |
|  | (Fafchamps et al., 2014) |
|  | (Fernald and Gunnar, 2009) |
|  | (Ferreira et al., 2009) |
|  | (Fintel and Pienaar, 2016) |
|  | (Fitzsimons and Mesnard, 2014) |
|  | (Galama et al., 2017) |
|  | (Galasso and Ravallion, 2004) |
|  | (Galiani and McEwan, 2013) |
|  | (Gao, 2018) |
|  | (Gao et al., 2019) |
|  | (Garcia-Verdu, 2002) |
|  | (Garganta et al., 2017) |
|  | (Gertler and Boyce, 2001) |
|  | (Gertler et al., 2012) |
|  | (Grogan and Summerfield, 2019) |
|  | (Handa et al., 2018) |
|  | (Handa et al., 2016) |
|  | (Hoddinott et al., 2017) |
|  | (Hoffmann, 2008) |
|  | (Karlan et al., 2014) |
|  | (Kaushal, 2013) |
|  | (Kertesi and Kezdi, 2014) |
|  | (Kilburn, Handa, Wrong outcome Angeles, Tsoka, & Mvula, 2018) Wrong outcome |
|  | (Kohler and Thornton, 2012) |
|  | (Kollamparambil et al., 2019) |
|  | (Kugler and Rojas, 2018) |
|  | (Larranaga et al., 2012) |
|  | (Lee et al., 2019) |
|  | (Levere, 2016) |
|  | (Lopez Boo and Creamer, 2019) |
|  | (Luseno et al., 2014) |
|  | (Magda et al., 2018) |
|  | (Maluccio and Flores, 2005) |
|  | (Maluccio, 2009) |
|  | (Martinez and Maia, 2018) |
|  | (Martorano and Sanfilippo, 2012) |
|  | (Miller, 2011) |
|  | (Miller et al., 2011) |
|  | (Mostert and Vall Castello, 2020) |
|  | (Natali et al., 2018) |
|  | (Nayab and Farooq, 2014) |
|  | (Okeke and Abubakar, 2020) |
|  | (Pace et al., 2019) |
|  | (Pais et al., 2017) |
|  | (Pak, 2020) |
|  | (Patel et al., 2015) |
|  | (Perova and Vakis, 2012) |
|  | (Pettifor et al., 2016) |
|  | (Pi Alperin, 2009) |
|  | (Ponce and Bedi, 2010) |
|  | (Rahman, 2014) Wrong outcome |
|  | (Robertson et al., 2012) |
|  | (Robertson et al., 2013) |
|  | (Robinson, 2012) |
|  | (Roy et al., 2017) |
|  | (Salehi-Isfahani and Mostafavi-Dehzooei, 2018) |
|  | (Schatz et al., 2012) |
|  | (Sedlmayr et al., 2020) |
|  | (Singh, 2019) |
|  | (Sulaiman et al., 2016) |
|  | (Szulc, 2012) |
|  | (Tagliati, 2019) |
|  | (Taylor et al., 2016) |
|  | (Tiwari, 2019) |
|  | (Unnikishnan and Imai, 2018) |
|  | (Urrea and Maldonado, 2011) |
|  | (Viegas Andrade, Chein, Perez Ribas, & Puig-Junoy, 2013) |
|  | (Waqas and Awan, 2018) |
|  | (Whetten et al., 2019) |
|  | (Yang, 2013) |
|  | (Daidone et al., 2019) |
| Wrong population (e.g. children, spillover to non-recipients) | (Abu-Hamad et al., 2014) |
|  | (Angelucci et al., 2018) |
|  | (Blattman and Dercon, 2018) |
|  | (Egger et al., 2019) |
|  | (Fernald et al., 2008) |
|  | (Ko, 2019) |
|  | (Manley et al., 2015) |
|  | (Ozer et al., 2009) |
|  | (Reis, 2010) |
|  | (Valadez-Martinez, 2016) |
| Wrong study design (e.g. quasi-experimental, case study, qualitative) | (Ayuku et al., 2014) |
|  | (Bando et al., 2016) |
|  | (Barham et al., 2018) |
|  | (Béné et al., 2012) |
|  | (Bonnerjee, 2017) |
|  | (Brewer et al., 1997) |
|  | (Case, 2001) |
|  | (Chen et al., 2019) |
|  | (Drucza, 2016) |
|  | (Eyal and Burns, 2019) |
|  | (Galiani et al., 2016) |
|  | (Gros et al., 2019) |
|  | (Hagen-Zanker et al., 2018) |
|  | (Hoddinott and Adato, 2010) |
|  | (Hoddinott and Wiesmann, 2010) |
|  | (Nalwanga et al., 2018) |
|  | (Ohrnberger et al., 2020) |
|  | (Ozer et al., 2011) |
|  | (Plagerson et al., 2011) |
|  | (Powell-Jackson et al., 2016) |
|  | (Salinas-Rodríguez et al., 2014) |
|  | (Shangani et al., 2017) |
|  | (Sugiyama and Hunter, 2020) |
|  | (Thompson, 2014) |
|  | (Zhang et al., 2019) |
| Ongoing evaluation | (Field and Maffioli, 2016) |
| Original study included | (Hong Mei and Jiang Tao, 2019) |
| Published paper included | (Haushofer and Shapiro, 2013) |
|  | (Haushofer et al., 2015) |
|  | (Paxson and Schady, 2010) |
| Relevant outcomes not reported | (Mills et al., 2018) |

**Supplementary Appendix S3.** **Characteristics of included studies**

| Program | | Study | Target Group (age^1^) | Country | Outcome | Transfer amount | Study type | Control group | Program  duration^2^ | Time to  follow-up | Key findings | |
| --- | --- | --- | --- | --- | --- | --- | --- | --- | --- | --- | --- | --- |
| Malawi Social Cash Transfer Program (UCT) | | Abdoulayi et al (2017) | Adolescents (13-19), adult caregivers (∅58) | Malawi | Depression (CES-D), stress (PSS) | US$3–7 per month, plus  US$1-2 per primary / secondary  school aged child | cRCT | Waitlist control | 24 months | Post-intervention | Decrease in depression, significant only for poorest 50% of recipients; significant reduction in caregiver stress | |
|  |  | Angeles et al., (2019) | Adolescents (13-19), adult caregivers (nr) | Malawi | Depression (CES-D), stress (PSS) | US$3–7 per month, plus  US$1-2 per primary / secondary  school aged child | cRCT | Waitlist control | 24 months | Post-intervention | Significant improvement in depression, particularly for adolescent girls; reduction in caregiver stress | |
| Zomba Cash Transfer Program (UCT, CCT) | | Baird, de Hoop, & Özler (2013) | Adolescents / young adults (13-22) | Malawi | Anxiety and Depression (GHQ-12) | US$4-10 per month to parents, US$1-5 per month to girls | cRCT | No transfer | 12 months | Post-intervention | Significant improvement during the program, particularly for unconditional transfers; effects for conditional transfer to those who were school dropouts at baseline not significant; significant effects disappear soon after program cessation | |
|  |  | Baird et al. (2015) | Adolescents / young adults (13-22) | Malawi | Anxiety and Depression (GHQ-12) | US$4-10 per month to parents, US$1-5 per month to girls | cRCT | No transfer | 24 months | 2 years | Positive effects have dissipated four years after program cessation | |
| Youth Opportunity Program (UCT) | | Blattman, Fiala & Martinez (2018) | Young adults (16-35) | Uganda | Depression (PHQ-9), stress (PSS) | US$384 in total | cRCT | No transfer | Lump sum | 9 years | Insignificant reduction in stress and depression, that were larger for women than men | |
| GiveDirectly Kenya (UCT) | | Haushofer & Shapiro (2016) | Adults (∅35) | Kenya | Depression (CES-D), Stress (PSS) | US$404 to US$1,525 PPP in total | cRCT | No transfer | 9 months | Post-intervention | Significant improvements in depression and stress, particularly for large transfers | |
|  |  | Haushofer & Shapiro (2018) | Adults (∅35) | Kenya | Depression (CES-D), Stress (PSS) | US$404 to US$1,525 PPP in total | cRCT | No transfer | 9 months | 2 years | Significant improvement in depression was maintained, stress reduction no longer significant | |
| Nairobi Microinsurance Project (UCT) | | Haushofer et al. (2020) | Adults (∅33) | Kenya | Depression (CES-D), Stress (PSS) | US$328 PPP in total per household; additional US$52 PPP for households with more than five dependents | RCT | No transfer | Lump sum | Post-intervention | No significant effects on stress and depression | |
| Jigisemejiri National Cash Transfer Program (UCT) | | Heath, Hidrobo, & Roy, (2020) | Male adult partners of recipients (∅44) | Mali | Stress (PSS) | US$18.02 per month | cRCT | Waitlist control | 12 months | Post-intervention | Significant reductions in stress, particularly for polygamous households | |
| Zambia Child Grant Programme (UCT) | | Hjelm, Handa, Hoop, & Palermo (2017) | Female adults (∅30) | Zambia | Stress (PSS) | US$11 per month per household | cRCT | Waitlist control | 36 months | Post-intervention | No significant effects on stress | |
| Multiple Category Cash Transfer Program (UCT) | | Hjelm, Handa, Hoop, & Palermo (2017) | Female adults (∅52) | Zambia | Stress (PSS) | US$11 per month per household | cRCT | Waitlist control | 36 months | Post-intervention | No significant effects on stress | |
| Cash Transfer for Orphans and Vulnerable Children (UCT) | | Kilburn et al. (2016) | Young adults (15-24) | Kenya | Depression (CES-D) | US$20 per month | cRCT | Waitlist control | 48 months | Post-intervention | Significant effects only for young men, particularly those aged 20-24 years and orphans | |
| HIV Prevention Trials Network 068 Study (CCT) | | Kilburn et al. (2019) | Young female adults (13-20) | South Africa | Depression (CES-D) | US$10 for adolescent per month, US$20 for guardian | RCT | No transfer | 36 months | Post-intervention | Significant effects only for recipients of the 50% poorest households | |
|  |  | Pettifor et al. (2016) | Young female adults (13-20) | South Africa | Anxiety and Depression (CES-D, CDI, CMAS) | US$10 for adolescent per month, US$20 for guardian | RCT | No transfer | 9 months | Post-intervention | No significant effects overall, borderline significant effects for poorest quintile | |
| Atención A Crisis (UCT) | | Macours et al. (2012) | Adult caregivers (∅40) | Nicaragua | Depression (CES-D) | US$145 per household per year plus US$200 lump sum | cRCT | No transfer | 9-12 months | Post-intervention | No significant effects on depression | |
|  |  | Macours & Vakis (2014) | Adult caregivers (∅40) | Nicaragua | Depression (CES-D) | US$145 per household per year plus US$200 lump sum | cRCT | No transfer | 9 months | Post-intervention & 2-year follow-up | No significant effects on depression | |
| Bono De Desarrollo Humano (UCT) | | Paxson & Schady (2010) | Female adult caregiver (∅23-24) | Ecuador | Depression (CES-D), Stress (PSS) | US$15 per month per family | cRCT | Waitlist control | 17 months | Post-intervention | Insignificant reduction on depression, significant increase in stress for poorest quartile | |
| Transfer Modality Research Initiative (UCT) | | Roy et al. (2019) | Male adult (nr) | Bangladesh | Depression (PHQ-9), Stress (PSS) | US$19 per month per household | cRCT | No transfer | 12 months | 4 years | Significant effects on depression and stress only for Cash + nutritional behavior intervention | |
|  | *^1^represents the age at transfer receipt, ^2^represents the duration of received payments at time of outcome measurement*  *Notes: nr = not reported, UCT = unconditional cash transfer, CCT = conditional cash transfer, RCT = randomized controlled trial, cRCT = cluster RCT* | | | | | | | | | | |  |

**Supplementary Appendix S4.** **R Code**

#Loading relevant packages
library(tidyverse)
library(pander)
library(robumeta)
library(metafor)
library(clubSandwich)
library(PublicationBias)

**##Step 1: Conducting RVE**

#a) Depression/Anxiety post-intervention

model1 <- robu(formula = cohens_d ~ 1, #Simple model, without covariates
 var.eff.size=v, #Effect size variance
 studynum = program, #Adjusting dependency at program level
 modelweights = "CORR", #Correlated effects model, i.e. overlapping sample
 rho = 0.8, #Assumed correlation between effect sizes
 small=TRUE, #applies the small sample correction to df
 data=depressionT1)
print(model1)

#Creating forestplot
forest.robu(model1, es.lab = "study", study.lab = "program",
 "Weight" = r.weights, #displays the weight that each study is given
 "Cohen's d [95%-CI]" = d_ci, #displays the effect size and 95%-CI
 "Measure" = measure) #Displays the measurement method used

#Sensitivity analysis for different rho-values
sensitivity(model1)

#b) Depression/Anxiety follow-up

model2 <- robu(formula = cohens_d ~ 1, var.eff.size=v, studynum = program,
 modelweights = "CORR", rho = 0.8, small=TRUE,
 data=depressionT2)
print(model2)

#Creating forestplot
forest.robu(model2, es.lab = "study", study.lab = "program", "Weight" = r.weights,
 "Cohen's d [95%-CI]" = d_ci, "Measure" = measure)

#Sensitivity analysis
sensitivity(model2)

#c) Stress post-intervention
model3 <- robu(formula = cohens_d ~ 1, var.eff.size=v, studynum = program,
 modelweights = "CORR", rho = 0.8, small=TRUE,
 data=stressT1)
print(model3)

#Creating forestplot
forest.robu(model3, es.lab = "study", study.lab = "program", "Weight" = r.weights,
 "Cohen's d [95%-CI]" = d_ci, "Measure" = measure)

#Sensitivity analysis
sensitivity(model3)

**##Step 2: Conducting multilevel meta-analysis**

#a) Depression/anxiety post-intervention
MLM_Model <- rma.mv(yi=cohens_d #Effect size
 , V= v #Effect size variance
 , data=depressionT1
 , random=list( ~1|study #Dependecy within studies
 , ~1|program) #Dependecy within programs
 , slab=outcome) #Labels for forest plot
print(MLM_Model)

forest(MLM_Model #Object containing estimates
 , showweights=TRUE #Displays inverse-variance weighting
 , cex=0.70 #Font size
 , main="MLM - Depression & Anxiety Post-intervention"
 , xlab="Cohen's d"
 , mlab="Grand Mean",
 slab = depressionT1$study)

#Calculate I-squared
#At study-level
I2study1 <- (MLM_Model$sigma2[1])/
 (MLM_Model$sigma2[1]+
 MLM_Model$sigma2[2]+
 mean(depressionT1$v))

I2study1

#At program-level
I2program1<-(MLM_Model$sigma2[2])/
 (MLM_Model$sigma2[1]+
 MLM_Model$sigma2[2]+
 mean(depressionT1b$v))

I2program1

#b) Depression/anxiety follow-up

MLM_Model2 <- rma.mv(yi=cohens_d
 , V= v
 , data=depressionT2
 , random=list( ~1|study
 , ~1|program)
 , slab=outcome)
summary(MLM_Model2)

forest(MLM_Model2
 , showweights=TRUE
 , cex=0.70
 , main="MLM - Depression & Anxiety Follow-up"
 , xlab="Cohen's d"
 , mlab="Grand Mean",
 slab = depressionT2$study)

#I-squared
I2es <- (MLM_Model2$sigma2[1])/
 (MLM_Model2$sigma2[1]+MLM_Model2$sigma2[2]+ mean(depressionT2$v))

I2es

I2program2<-(MLM_Model2$sigma2[2])/
 (MLM_Model2$sigma2[1]+
 MLM_Model2$sigma2[2]+
 mean(stressT1$v))

I2program2

#c) Stress post-intervention
MLM_Model3 <- rma.mv(yi=cohens_d
 , V= v
 , data=stressT1
 , random=list( ~1|study
 , ~1|program)
 , slab=outcome)
summary(MLM_Model3)

forest(MLM_Model3
 , showweights=TRUE
 , cex=0.70 #Font size
 , main="MLM - Stress"
 , xlab="Cohen's d"
 , mlab="Grand Mean",
 slab = stressT1$study)

#I-squared
I2study3 <- (MLM_Model3$sigma2[1])/
 (MLM_Model3$sigma2[1]+MLM_Model3$sigma2[2]+ mean(stressT1$v))

I2study3

I2program3<-(MLM_Model3$sigma2[2])/
 (MLM_Model3$sigma2[1]+
 MLM_Model3$sigma2[2]+
 mean(stressT1$v))

I2program3

**#Step 3: Assessing publication bias**

#Drawing funnelplot for depression/anxiety post-intervention
funnel(MLM_Model,
 level=c(90, 95, 99), #Contour-enhanced at different significance level
 shade=c("white", "gray55", "gray75"),
 refline=0,
 xlab = "Cohen's d values",
 legend=TRUE)

#Egger's regression test
regtest(depressionT1$cohens_d, depressionT1$v, model="rma")

#Testing robustness of findings to potential publication bias
#Assumption: significant positive findings 40-times more likely to be published
corrected_meta(yi = depressionT1$cohens_d, #effect sizes
 vi = depressionT1$v, #variances
 eta = 40, #40-times more likely to be published
 model = "robust", #RVE model
 clustervar = depressionT1$program,#SEs adjusted at program-level
 favor.positive = FALSE) #publication bias would favor negative effects

Sources: (Fisher and Tipton, 2015; Mathur and Vanderweele, 2019; Nye, 2019; Viechtbauer, 2010)

**Supplementary Appendix S5. Robustness tests**

Analysis conducted using R package *metafor* (Viechtbauer, 2010) and *robumeta* (Fisher and Tipton, 2015)*.*

Table S5.1. **Sensitivity Analysis RVE meta-analysis depression/anxiety post-intervention**

|  | **Rho=0** | **Rho=0.2** | **Rho=0.4** | **Rho=0.6** | **Rho=0.8** | **Rho=1** |
| --- | --- | --- | --- | --- | --- | --- |
| **Coefficient** | -0.10193 | -0.10193 | -0.10192 | -0.10192 | -0.10191 | -0.1019 |
| **Std. Error** | 0.02053 | 0.02052 | 0.02050 | 0.02048 | 0.02046 | 0.0204 |

Table S5.2. **Sensitivity Analysis RVE meta-analysis depression/anxiety at follow-up**

|  | **Rho=0** | **Rho=0.2** | **Rho=0.4** | **Rho=0.6** | **Rho=0.8** | **Rho=1** |
| --- | --- | --- | --- | --- | --- | --- |
| **Coefficient** | -0.050933 | -0.050907 | -0.050881 | -0.050855 | -0.050829 | -0.050804 |
| **Std. Error** | 0.026465 | 0.026478 | 0.026491 | 0.026504 | 0.026516 | 0.026528 |

Table S5.3. **Sensitivity Analysis RVE meta-analysis stress post-intervention**

|  | **Rho=0** | **Rho=0.2** | **Rho=0.4** | **Rho=0.6** | **Rho=0.8** | **Rho=1** |
| --- | --- | --- | --- | --- | --- | --- |
| **Coefficient** | -0.1023 | -0.1023 | -0.1023 | -0.1023 | -0.1023 | -0.1023 |
| **Std. Error** | 0.0895 | 0.0895 | 0.0895 | 0.0895 | 0.0895 | 0.0895 |

Table S5.4. **Sensitivity Analysis RVE meta-regression**

|  | **Rho=0** | **Rho=0.2** | **Rho=0.4** | **Rho=0.6** | **Rho=0.8** | **Rho=1** |
| --- | --- | --- | --- | --- | --- | --- |
| **Coefficient** | -0.1352 | -0.1352 | -0.1352 | -0.1352 | -0.1352 | -0.1352 |
| **Std. Error** | 0.0109 | 0.0109 | 0.0109 | 0.0109 | 0.0109 | 0.0109 |
| **Condition Coefficient** | 0.1025 | 0.1025 | 0.1025 | 0.1025 | 0.1025 | 0.1025 |
| **Std. Error** | 0.0117 | 0.0117 | 0.0117 | 0.0117 | 0.0117 | 0.0117 |

Supplementary Figure S5.1 **MLMA: depression/anxiety post-intervention**

**
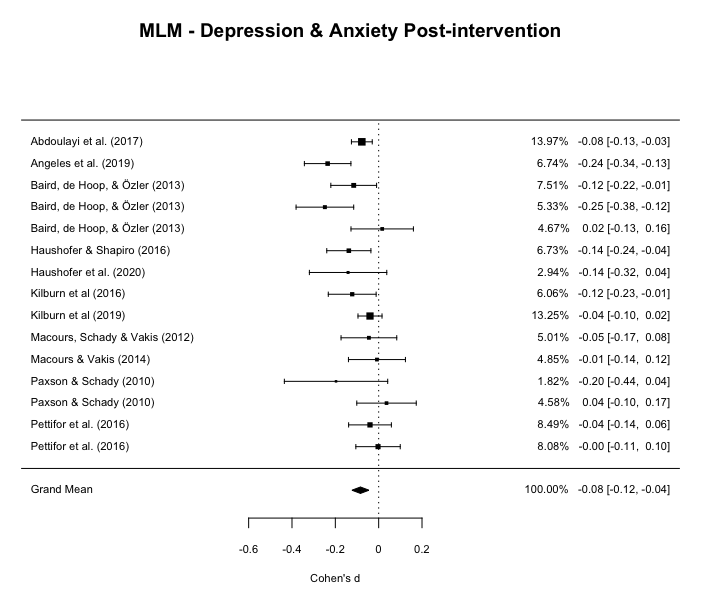
**

Supplementary Figure S5.2 **MLMA: depression/anxiety follow-up**

**
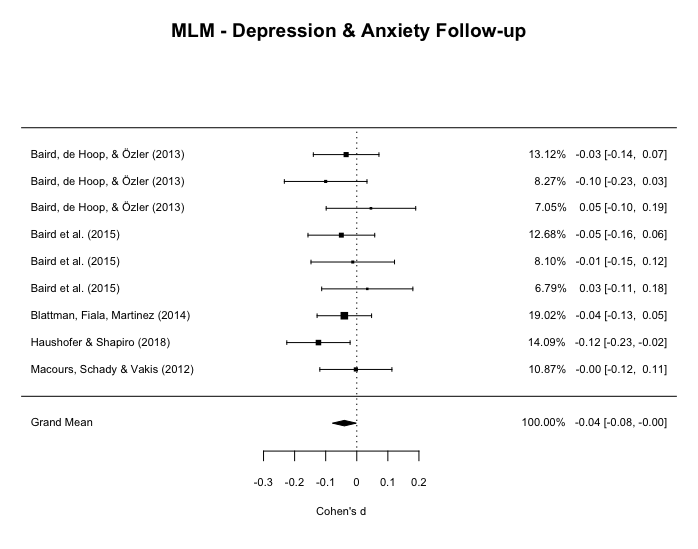
**

Supplementary Figure S5.3. **MLMA: stress post-intervention**


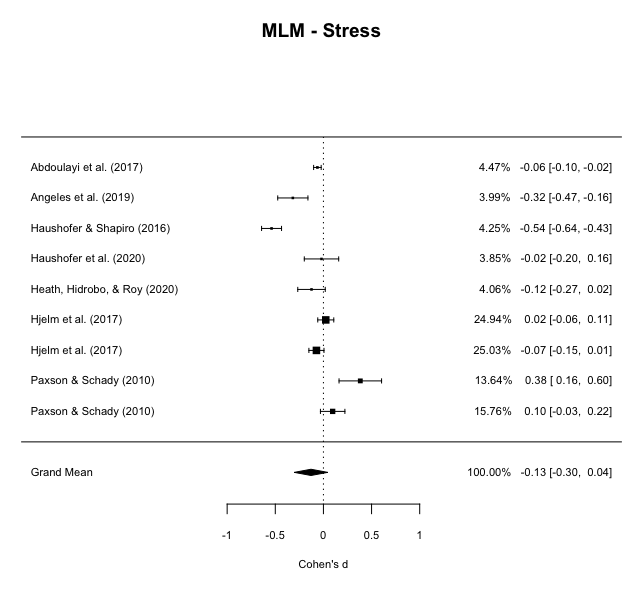


**References:**

Abu-Hamad, B., Jones, N., Pereznieto, P., 2014. Tackling children’s economic and psychosocial vulnerabilities synergistically: How well is the Palestinian National Cash Transfer Programme serving Gazan children? Spec. Issue Econ. Dimens. child Prot. well-being. 47, 121–135. https://doi.org/http://dx.doi.org/10.1016/j.childyouth.2014.09.009

Aker, J.C., 2017. Comparing Cash and Voucher Transfers in a Humanitarian Context: Evidence from the Democratic Republic of Congo. World Bank Econ. Rev. 31, 44–70.

Akresh, R., de Walque, D., Kazianga, H., 2012. Alternative Cash Transfer Delivery Mechanisms: Impacts on Routine Preventative Health Clinic Visits in Burkina Faso (No. 17785), NBER Working Paper.

Alcazar, L., Balarin, M., Espinoza, K., 2016. Impacts of the Peruvian Conditional Cash Transfer Program on Women’s Empowerment: A Quantitative and Qualitative Approach, PEP-PMMA, Working Papers PMMA. PEP-PMMA, Working Papers PMMA, Unlisted; Unlisted; Unlisted.

Almås, I., Armand, A., Attanasio, O., Carneiro, P., 2018. Measuring and Changing Control: Women’s Empowerment and Targeted Transfers. Econ. J. 128, F609–F639. https://doi.org/10.1111/ecoj.12517

Amarante, V., Manacorda, M., Miguel, E., Vigorito, A., 2016. Do cash transfers improve birth outcomes? Evidence from matched vital statistics, and program and social security data. Am. Econ. J. Econ. Policy 8, 1–43. https://doi.org/10.1257/pol.20140344

Amin, R., Becker, S., Bayes, A., 1998. NGO-promoted microcredit programs and women’s empowerment in rural Bangladesh: quantitative and qualitative evidence. J. Dev. Areas 32, 221–236.

Andrew, A., Attanasio, O., Fitzsimons, E., Grantham-McGregor, S., Meghir, C., Rubio-Codina, M., 2018. Impacts 2 years after a Scalable Early Childhood Development Intervention to Increase Psychosocial Stimulation in the Home: A Follow-up of a Cluster Randomised Controlled Trial in Colombia. PLoS Med. 15, e1002556.

Angelucci, M., De Giorgi, G., Rasul, I., 2018. Consumption and Investment in Resource Pooling Family Networks. Econ. J. 128, 2613–2651. https://doi.org/10.1111/ecoj.12534

Attah, R., Barca, V., Kardan, A., MacAuslan, I., Merttens, F., Pellerano, L., 2016. Can Social Protection Affect Psychosocial Wellbeing and Why Does This Matter? Lessons from Cash Transfers in Sub-Saharan Africa. J. Dev. Stud. 52, 1115–1131.

Attanasio, O., Mesnard, A., 2006. The Impact of a Conditional Cash Transfer Programme on Consumption in Colombia. Fisc. Stud. 27, 421–442.

Ayuku, D., Embleton, L., Koech, J., Atwoli, L., Hu, L.Y., Ayaya, S., Hogan, J., Nyandiko, W., Vreeman, R., Kamanda, A., Braitstein, P., 2014. The government of Kenya cash transfer for orphaned and vulnerable children: cross-sectional comparison of household and individual characteristics of those with and without. BMC Int. Health Hum. Rights 14.

Bagolin, I.P., 2017. The Impact of Cash Transfer Programs on Brazilian’s Well-Being. Int. J. Soc. Econ. 44, 1106–1118.

Baird, S., Chirwa, E., de Hoop, J., Özler, B., 2013a. Girl Power: Cash Transfers and Adolescent Welfare. Evidence from a Cluster-Randomized Experiment in Malawi (No. 19479), NBER Working Paper Series. National Bureau of Economic Research, Inc, NBER Working Papers: 19479.

Baird, S., Ferreira, F.H.G., Özler, B., Woolcock, M., 2013b. Relative Effectiveness of Conditional and Unconditional Cash Transfers for Schooling Outcomes in Developing Countries: A Systematic Review. Campbell Syst. Rev. 9, 1–124. https://doi.org/10.4073/csr.2013.8

Baird, S., McIntosh, C., Özler, B., 2011. Cash or Condition? Evidence from a Cash Transfer Experiment. Q. J. Econ. 126, 1709–1753.

Bando, R., Galiani, S., Gertler, P., 2016. The Effects of Non-Contributory Pensions on Material and Subjective Well Being (No. 22995), NBER Working Paper Series. National Bureau of Economic Research, Inc, NBER Working Papers: 22995.

Banerjee, A., Duflo, E., Chattopadhyay, R., Shapiro, J., 2011. Targeting the Hard-Core Poor: An Impact Assessment.

Banerjee, A., Karlan, D., Osei, R., Thuysbaert, B., Udry, C., 2017. Graduation From Ultra Poverty In Ghana, 3ie Grantee Final Report.

Banerjee, A. V., Karlan, D., Osei, R.D., Trachtman, H., Udry, C., 2018. Unpacking a Multi-Faceted Program to Build Sustainable Income for the Very Poor. SSRN Electron. J. https://doi.org/10.2139/ssrn.3127053

Barham, T., Macours, K., Maluccio, J., 2018. Experimental Evidence of Exposure to a Conditional Cash Transfer During Early Teenage Years: Young Women’s Fertility and Labor Market Outcomes. CEPR Discuss. Pap.

Barrera-Osorio, F., Bertrand, M., Linden, L.L., Perez-Calle, F., 2008. Conditional Cash Transfers in Education Design Features, Peer and Sibling Effects Evidence from a Randomized Experiment in Colombia (No. 13890), NBER Working Paper Series. National Bureau of Economic Research, Inc, NBER Working Papers: 13890.

Barrera-Osorio, F., Linden, L.L., Saavedra, J., 2017. Medium- and Long-Term Educational Consequences of Alternative Conditional Cash Transfer Designs: Experimental Evidence from Colombia (No. 23275), NBER Working Paper Series. National Bureau of Economic Research, Inc, NBER Working Papers: 23275, Unlisted; Unlisted; Unlisted.

Barrientos, A., Villa, J.M., 2015. Antipoverty Transfers and Labour Market Outcomes: Regression Discontinuity Design Findings. J. Dev. Stud. 51, 1224–1240.

Bastagli, F., Hagen-Zanker, J., Harman, L., Barca, V., Sturge, G., Schmidt, T., Pellerano, L., 2016. Cash transfers: what does the evidence say? A rigorous review of programme impact and of the role of design and implementation features. ODI Rep. 1–300. https://doi.org/DOI: 10.13140/RG.2.2.29336.39687

Behrman, J.R., Gallardo-Garcia, J., Parker, S.W., Todd, P.E., Velez-Grajales, V., 2012. Are Conditional Cash Transfers Effective in Urban Areas? Evidence from Mexico. Educ. Econ. 20, 233–259.

Behrman, J.R., Parker, S.W., 2011. The Impact of the PROGRESA/Oportunidades Conditional Cash Transfer Program on Health and Related Outcomes for the Aging in Mexico. Penn Inst. Econ. Res. Dep. Econ. Univ. Pennsylvania, PIER Work. Pap. Arch. 2011, 25 pp.

Behrman, J.R., Parker, S.W., 2010. The Impacts of Conditional Cash Transfer Programs on Education, Conditional Cash Transfers in Latin America. 2010, pp. 191-211. Baltimore: Johns Hopkins University Press; Washington, D.C.: International Food Policy Research Institute.

Behrman, J.R., Parker, S.W., Todd, P.E., 2009. Medium-Term Impacts of the Oportunidades Conditional Cash-Transfer Program on Rural Youth in Mexico, in: Klasen, S., Nowak-Lehmann, F. (Eds.), Poverty, Inequality, and Policy in Latin America. CESifo Seminar Series. Cambridge and London: MIT Press, pp. 219–70.

Béné, C., Devereux, S., Sabates-Wheeler, R., 2012. Shocks and social protection in the Horn of Africa: analysis from the Productive Safety Net programme in Ethiopia (No. 395), IDS Working Papers. https://doi.org/10.1111/j.2040-0209.2012.00395.x

Benedetti, F., Ibarraran, P., McEwan, P.J., 2016. Do Education and Health Conditions Matter in a Large Cash Transfer? Evidence from a Honduran Experiment. Econ. Dev. Cult. Change 64, 759–793.

Berniell, I., de la Mata, D., Machado, M.P., 2014. The Impact of a Permanent Income Shock on the Situation of Women in the Household: the case of a pension reform in Argentina (No. 148), CEPR Discussion Papers. C.E.P.R. Discussion Papers, CEPR Discussion Papers: 10256, Unlisted; Unlisted; Unlisted.

Bhanot, S.P., Han, J., Jang, S.P., 2018. Workfare, wellbeing and consumption: Evidence from a field experiment with Kenya’s urban poor. J. Econ. Behav. Organ. 149, 372–388.

Blattman, C., Annan, J., 2015. Can employment reduce lawlessness and rebellion? A field experiment with high-risk men in a Fragile State (No. 21289), NBER Working Paper. https://doi.org/10.1017/S0003055415000520

Blattman, C., Annan, J., 2011. Reintegrating and Employing High-Risk Youth in Liberia: Lessons from a randomized evaluation of a Landmine Action and agricultural training program for ex-combatants, Evidence from Randomized Evaluations of Peacebuilding in Liberia: Policy Report.

Blattman, C., Dercon, S., 2018. The impacts of industrial and entrepreneurial work on income and health: Experimental evidence from Ethiopia. Am. Econ. J. Appl. Econ. 10, 1–38. https://doi.org/10.1257/app.20170173

Blattman, C., Fiala, N., Martinez, S., 2014. Generating skilled self-employment in developing countries: Experimental evidence from Uganda. Q. J. Econ. 697–752. https://doi.org/10.1093/qje/qjt057

Blattman, C., Green, E.P., Jamison, J., Christian Lehmann, M., Annan, J., 2016. The returns to microenterprise support among the ultrapoor: A field experiment in postwar Uganda (No. 21310), NBER Working Paper. https://doi.org/10.1257/app.20150023

Bobonis, G., 2011. The Impact of Conditional Cash Transfers on Marriage and Divorce. Econ. Dev. Cult. Change 59, 281–312.

Bobonis, G., Castro, R., Gonzalez-Brenes, M., 2013. Public Transfers and Domestic Violence: The Roles of Private Information and Spousal Control. Am. Econ. J. Econ. Policy 5, 179–205.

Bonnerjee, A., 2017. Piloting Social Protection in Chin State, Myanmar: Challenges and Opportunities within a Context of Fragility. Glob. Soc. Policy 17, 375–380.

Brenes-Camacho, G., 2011. Favourable changes in economic well-being and self-rated health among the elderly. Soc. Sci. Med. 72, 1228–1235.

Brewer, B., MacPherson, S., Wilding, P., Huque, A.S., 1997. Poverty and Social Security, in: Tao Lai Po-wah, J. (Ed.), Social Policy in Hong Kong. Cheltenham, U.K. and Lyme, N.H.: Elgar; distributed by American International Distribution Corporation, Williston, Vt., pp. 72–94.

Buller, A.M., Hidrobo, M., Peterman, A., Heise, L., 2016. The way to a man’s heart is through his stomach?: A mixed methods study on causal mechanisms through which cash and in-kind food transfers decreased intimate partner violence. BMC Public Health 16.

Burmaster, K.B., Landefeld, J.C., Rehkopf, D.H., Lahiff, M., Sokal-Gutierrez, K., Adler-Milstein, S., Fernald, L., 2015. Impact of a private sector living wage intervention on depressive symptoms among apparel workers in the Dominican Republic: a quasi-experimental study. BMJ Open 5, e007336.

Cahyadi, N., Hanna, R., Olken, B.A., Prima, R.A., Satriawan, E., Syamsulhakim, E., 2018. Cumulative Impacts of Conditional Cash Transfer Programs: Experimental Evidence from Indonesia (No. 24670), NBER Working Paper Series. National Bureau of Economic Research, Inc, NBER Working Papers: 24670, Unlisted; Unlisted; Unlisted; Unlisted; Unlisted; Unlisted.

Case, A., 2001. Health, Income and Economic Development (No. 207), Research Program in Development Studies.

Chen, X., Wang, T., Busch, S.H., 2019. Does money relieve depression? Evidence from social pension expansions in China. Soc. Sci. Med. 220, 411–420.

Christian, C., Hensel, L., Roth, C., 2019. Income Shocks and Suicides: Causal Evidence from Indonesia. Rev. Econ. Stat. 101, 905–920.

Coetzee, M., 2013. Finding the Benefits: Estimating the impact of the South African Child Support Grant. South African J. Econ. 81, 427–450.

Crea, T.M., Reynolds, A.D., Sinha, A., Eaton, J.W., Robertson, L.A., Mushati, P., Dumba, L., Mavise, G., Makoni, J.C., Schumacher, C.M., Nyamukapa, C.A., Gregson, S., 2015. Effects of cash transfers on children’s health and social protection in sub-Saharan Africa: differences in outcomes based on orphan status and household assets. BMC Public Health 15.

Cunha, J.M., Giorgi, G. De, Jayachandran, S., 2019. The Price Effects of Cash Versus In-Kind Transfers. Rev. Econ. Stud. 86, 240–281.

D’Aoust, O., Sterck, O., Verwimp, P., 2013. Buying Peace: The Mirage of Demobilizing Rebels (No. 2013009), IRES Discussion Papers. Universite catholique de Louvain, Institut de Recherches Economiques et Sociales (IRES), Discussion Papers (IRES - Institut de Recherches Economiques et Sociales): 2013009, Universite Libre de Bruxelles (SBS-EM, ECARES) and FNRS; UNIVERSITE CATHOLIQUE DE LOUVAIN, Institut de Recherches Economiques et Sociales (IRES); Universite libre de Bruxelles (SBS-EM, ECARES, Centre Emile Bernheim).

Daidone, S., Davis, B., Handa, S., Winters, P., 2019. The Household and Individual-Level Productive Impacts of Cash Transfer Programs in Sub-Saharan Africa. Am. J. Agric. Econ. 101, 1401–1431.

Dake, F., Natali, L., Angeles, G., de Hoop, J., Handa, S., Peterman, A., 2018. Cash Transfers, Early Marriage, and Fertility in Malawi and Zambia. Stud. Fam. Plann. 49, 295–317.

de Oliveira, P.R., Kassouf, A.L., de Aquino, J.M., 2017. Cash Transfers to the Elderly and Its Spillover Effects: Evidences from a Non-contributory Program in Brazil. J. Econ. Stud. 44, 183–205.

Delgado, O.A.S., Kadelbach, V., Mata Mata, L., 2018. Effects of Conditional Cash Transfers (CCT) in Anti-poverty Programs: An Empirical Approach with Panel Data for the Mexican Case of PROSPERA-Oportunidades (2002-2012). Economies 6, 1–13.

Ding, Y., 2017. Personal Life Satisfaction of China’s Rural Elderly: Effect of the New Rural Pension Programme. J. Int. Dev. 29, 52–66.

Drucza, K., 2016. Cash Transfers in Nepal: Do They Contribute to Social Inclusion? Oxford Dev. Stud. 44, 49–69.

Edmonds, E. V, Schady, N., 2012. Poverty Alleviation and Child Labor. Am. Econ. J. Econ. Policy 4, 100–124. https://doi.org/http://dx.doi.org/10.1257/pol.4.4.100

Egger, D., Haushofer, J., Miguel, E., Niehaus, P., Walker, M., 2019. General Equilibrium Effects of Cash Transfers: Experimental Evidence from Kenya (No. 26600), NBER Working Paper. https://doi.org/10.3386/w26600

EPOC, 2012. LMIC Filters, Cochrane Effective Practice and Organisation of Care Group [WWW Document]. URL https://epoc.cochrane.org/lmic-filters (accessed 3.21.20).

Eremina, S.L., Sun, F, Kudelina, O. V, Babaeva, F.G., Eremina, S.L., Sun, Fu, Kudelina, O. V, Babaeva, F.G., 2016. Russian National Social Security System as the Condition of Pensioners’ Well-Being, in: WELLSO 2015 - II International Scientific Symposium on Lifelong Wellbeing in the World. pp. 215–226.

Evans, D.K., Hausladen, S., Kosec, K., Reese, N., 2014. Community-Based Conditional Cash Transfers in Tanzania: Results from a Randomized Trial, World Bank Studies. World Bank Publications, Washington, DC, US.

Eyal, K., Burns, J., 2019. The parent trap: cash transfers and the intergenerational transmission of depressive symptoms in South Africa. World Dev. 117, 211–229. https://doi.org/http://dx.doi.org/10.1016/j.worlddev.2019.01.014

Fafchamps, M., McKenzie, D., Quinn, S., Woodruff, C., 2014. Microenterprise growth and the flypaper effect: Evidence from a randomized experiment in Ghana. J. Dev. Econ. 106, 211–226. https://doi.org/10.1016/j.jdeveco.2013.09.010

Fernald, L., Gertler, P.J., Neufeld, L.M., 2008. Role of cash in conditional cash transfer programmes for child health, growth, and development: an analysis of Mexico’s Oportunidades. Lancet 371, 828–837. https://doi.org/10.1016/S0140-6736(08)60382-7

Fernald, L., Gunnar, M.R., 2009. Poverty-alleviation program participation and salivary cortisol in very low-income children. Soc. Sci. Med. 68, 2180–2189. https://doi.org/http://dx.doi.org/10.1016/j.socscimed.2009.03.032

Ferreira, F.H.G., Filmer, D., Schady, N., Bandyopadhyay, S., 2009. Own and Sibling Effects of Conditional Cash Transfer Programs: Theory and Evidence from Cambodia, Policy Research Working Paper 5001. The World Bank.

Field, E., Maffioli, E., 2016. The Health Impacts and Effective Delivery of Maternal Cash Transfers in Myanmar [WWW Document]. Abdul Latif Poverty Action Lab. URL https://www.povertyactionlab.org/evaluation/health-impacts-and-effective-delivery-maternal-cash-transfers-myanmar

Fintel, D. von, Pienaar, L., 2016. Small-scale farming and food security: the enabling role of cash transfers in South Africa’s former homelands. Work. Pap. - Econ. Res. South. Africa 1–33.

Fisher, Z., Tipton, E., 2015. robumeta: An R-package for robust variance estimation in meta-analysis.

Fitzsimons, E., Mesnard, A., 2014. Can Conditional Cash Transfers Compensate for a Father’s Absence? World Bank Econ. Rev. 28, 467–491.

Galama, T.J., Morgan, R., Saavedra, J.E., 2017. Wealthier, Happier and More Self-Sufficient: When Anti-Poverty Programs Improve Economic and Subjective Wellbeing at a Reduced Cost to Taxpayers (No. 24090), NBER Working Paper. National Bureau of Economic Research, Inc, NBER Working Papers: 24090, Unlisted; Unlisted; Unlisted.

Galasso, E., Ravallion, M., 2004. Social Protection in a Crisis: Argentina’s Plan Jefes y Jefas. World Bank Econ. Rev. 18, 367–399.

Galiani, S., Gertler, P., Bando, R., 2016. Non-contributory pensions. Labour Econ. 38, 47–58.

Galiani, S., McEwan, P.J., 2013. The heterogeneous impact of conditional cash transfers. J. Public Econ. 103, 85–96. https://doi.org/10.1016/j.jpubeco.2013.04.004

Gao, Q., 2018. Welfare, Work, and Poverty: Social Assistance in China. https://doi.org/10.1093/oso/9780190218133.001.0001

Gao, Q., Zhang, Y., Zhai, F., 2019. Social Assistance in China: Impact Evaluation and Policy Implications. China An Int. J. 17, 3–9.

Garcia-Verdu, R., 2002. Evaluation of Conditional Income Support Programs: The Case of Mexico’s Progresa. Univ. Chicago.

Garganta, S., Gasparini, L., Marchionni, M., 2017. Cash Transfers and Female Labor Force Participation: The Case of AUH in Argentina. IZA J. Labor Policy 6.

Gertler, P.J., Boyce, S., 2001. An Experiment in Incentive-Based Welfare : The Impact of PROGESA on Health in Mexico.

Gertler, P.J., Martinez, S.W., Rubio-Codina, M., 2012. Investing cash transfers to raise long-term living standards. Am. Econ. J. Appl. Econ. 4, 1–32. https://doi.org/10.1257/app.4.1.164

Green, E.P., Blattman, C., Jamison, J., Annan, J., 2016. Does poverty alleviation decrease depression symptoms in post-conflict settings? A cluster-randomized trial of microenterprise assistance in Northern Uganda. Glob. Ment. Heal. 3. https://doi.org/10.1017/gmh.2015.28

Grogan, L., Summerfield, F., 2019. Government Transfers, Work, and Wellbeing: Evidence from the Russian Old-Age Pension. J. Popul. Econ. 32, 1247–1292.

Gros, C, Bailey, M, Schwager, S, Hassan, A, Zingg, R, Uddin, M M, Shahjahan, M, Islam, H, Lux, S, Jaime, C, de Perez, E C, Gros, Clemens, Bailey, Meghan, Schwager, Saroja, Hassan, Ahmadul, Zingg, Raymond, Uddin, Muhammad Mamtaz, Shahjahan, Mohammad, Islam, Hasibul, Lux, Stefanie, Jaime, Catalina, de Perez, Erin Coughlan, 2019. Household-level effects of providing forecast-based cash in anticipation of extreme weather events: Quasi-experimental evidence from humanitarian interventions in the 2017 floods in Bangladesh. Int. J. Disaster Risk Reduct. 41. https://doi.org/https://doi.org/10.1016/j.ijdrr.2019.101275

Hagen-Zanker, J, Ulrichs, M, Holmes, R, Hagen-Zanker, Jessica, Ulrichs, Martina, Holmes, Rebecca, 2018. What are the effects of cash transfers for refugees in the context of protracted displacement? Findings from Jordan. Int. Soc. Secur. Rev. 71, 57–77.

Handa, S., Natali, L., Seidenfeld, D., Tembo, G., Davis, B., 2018. Can Unconditional Cash Transfers Raise Long-Term Living Standards? Evidence from Zambia. J. Dev. Econ. 133, 42–65. https://doi.org/https://doi.org/10.1016/j.jdeveco.2018.01.008

Handa, S., Seidenfeld, D., Davis, B., Tembo, G., 2016. The Social and Productive Impacts of Zambia’s Child Grant. J. Policy Anal. Manag. 35, 357–387.

Haushofer, J., Reisinger, J., Shapiro, J., 2015. Your Gain Is My Pain: Negative Psychological Externalities of Cash Transfers.

Haushofer, J., Shapiro, J., 2013. Household Response to Income Changes: Evidence from an Unconditional Cash Transfer Program in Kenya. Work. Pap.

Hoddinott, J., Adato, M., 2010. Nutrition and Conditional Cash Transfer Programs, Conditional Cash Transfers in Latin America. 2010, pp. 231-57. Baltimore: Johns Hopkins University Press; Washington, D.C.: International Food Policy Research Institute.

Hoddinott, J., Ahmed, I., Ahmed, A., Roy, S., 2017. Behavior change communication activities improve infant and young child nutrition knowledge and practice of neighboring nonparticipants in a cluster-randomized trial in rural Bangladesh. PLoS One 12, 1–13. https://doi.org/10.1371/journal.pone.0179866

Hoddinott, J., Wiesmann, D., 2010. The Impact of Conditional Cash Transfer Programs on Food Consumption, in: Adato, M., Hoddinott, J. (Eds.), Conditional Cash Transfers in Latin America. Johns Hopkins University Press, Baltimore, pp. 258–83.

Hoffmann, V.E., 2008. Essays on Poverty Alleviation and Health Promotion in East Africa. Cornell Univ.

Hong Mei, W., Jiang Tao, L., 2019. The short-term impact of Unconditional Cash Transfers: a replication study of a randomized controlled trial in Kenya. Spec. Issue Replications Res. Financ. Serv. poor 11, 391–408.

Karimli, L, Ssewamala, F M, Neilands, T B, Wells, C R, Bermudez, L G, Karimli, Leyla, Ssewamala, Fred M, Neilands, Torsten B, Wells, Christine R, Bermudez, Laura Gauer, 2019. Poverty, economic strengthening, and mental health among AIDS orphaned children in Uganda: Mediation model in a randomized clinical trial. Soc. Sci. Med. 228, 17–24.

Karlan, D., Osei, R., Osei-Akoto, I., Udry, C., 2014. Agricultural decisions after relaxing credit and risk constraints. Q. J. Econ. 129, 597–652. https://doi.org/10.1093/qje/qju002

Kaushal, N., 2013. How Public Pension affects Elderly Labor Supply and Well-being: Evidence from India (No. 19088), NBER Working Paper. National Bureau of Economic Research, Inc, NBER Working Papers: 19088, Unlisted.

Kertesi, G., Kezdi, G., 2014. The Kindergarten Attendance Allowance in Hungary: Evaluation of a Conditional Cash Transfer Program. Acta Oeconomica 64, 27–49.

Kilburn, K., Handa, S., Angeles, G., Tsoka, M., Mvula, P., 2018. Paying for Happiness: Experimental Results from a Large Cash Transfer Program in Malawi. J. Policy Anal. Manag. 37, 331–356.

Ko, H., 2019. Intergenerational effects of old-age pension on children’s health and well-being. Int. J. Health Plann. Manage. 34, e1208–e1214.

Kohler, H.-P., Thornton, R.L., 2012. Conditional Cash Transfers and HIV/AIDS Prevention: Unconditionally Promising? World Bank Econ. Rev. 26, 165–190.

Kollamparambil, U, Etinzock, M N, Kollamparambil, Umakrishnan, Etinzock, Mfongeh N, 2019. Subjective well-being impact of old age pension in South Africa: A difference in difference analysis across the gender divide. South African J. Econ. Manag. Sci. 22, a2996. https://doi.org/10.4102/sajems.v22i1.2996

Kugler, A.D., Rojas, I., 2018. Do CCTs Improve Employment and Earnings in the Very Long-Term? Evidence from Mexico (No. 24248), NBER Working Paper. National Bureau of Economic Research, Inc, NBER Working Papers: 24248, Unlisted; Unlisted.

Larranaga, O, Contreras, D, Ruiz-Tagle, J, Larranaga, Osvaldo, Contreras, Dante, Ruiz-Tagle, Jaime, 2012. Impact Evaluation of Chile Solidario: Lessons and Policy Recommendations. J. Lat. Am. Stud. 44, 347–372.

Lee, S., Ku, I., Shon, B., 2019. The Effects of Old-Age Public Transfer on the Well-Being of Older Adults: The Case of Social Pension in South Korea. Journals Gerontol. Soc. Sci. 74, 506–515. https://doi.org/doi:10.1093/geronb/gbx104

Levere, M., 2016. Essays on Early Childhood and Adolescence. Univ. California, San Diego, 2016.

Lopez Boo, F., Creamer, J., 2019. Cash, Conditions, and Child Development: Experimental Evidence from a Cash Transfer Program in Honduras. Econ. J. Lat. Am. Caribb. Econ. Assoc. 19, 169–196.

Luseno, W.K., Singh, K., Handa, S., Suchindran, C., 2014. A Multilevel Analysis of the Effect of Malawi’s Social Cash Transfer Pilot Scheme on School-Age Children’s Health. Health Policy Plan. 29, 421–432.

Macours, K., Premand, P., Vakis, R., 2012. Transfers, Diversification and Household Risk Strategies: Experimental evidence with lessons for climate change adaptation (No. 6053), Policy Research Working Paper.

Magda, I., Kielczewska, A., Brandt, N., 2018. The “family 500+” child allowance and female labour supply in Poland (No. 1481), OECD Economics Department Working Papers. OECD Publishing, OECD Economics Department Working Papers: 1481.

Maluccio, J.A., 2009. Education and Child Labor: Experimental Evidence from a Nicaraguan Conditional Cash Transfer Program, in: Orazem, P.F., Sedlacek, G., Tzannatos, Z. (Eds.), Child Labor and Education in Latin America: An Economic Perspective. Houndmills, U.K. and New York: Palgrave Macmillan, pp. 187–204.

Maluccio, J.A., Flores, R., 2005. Impact Evaluation of a Conditional Cash Transfer Program: The Nicaraguan Red de Proteccion Social, IFPRI Research Report 141. Research Report 141. Washington, D.C.: International Food Policy Research Institute, Washington, D.C.

Manley, J., Fernald, L., Gertler, P., 2015. Wealthy, healthy, and wise: does money compensate for being born into difficult conditions? Appl. Econ. Lett. 22, 121–126. https://doi.org/10.1080/13504851.2014.929618

Martinez, D.M., Maia, A.G., 2018. The impacts of cash transfers on subjective wellbeing and poverty: The case of Colombia. J. Fam. Econ. Issues, Alternative Lifestyles, Lifestyles 39, 616–633. https://doi.org/https://doi.org/10.1007/s10834-018-9585-4

Martorano, B., Sanfilippo, M., 2012. Innovative Features in Poverty Reduction Programmes: An Impact Evaluation of Chile Solidario on Households and Children. J. Int. Dev. 24, 1030–1041.

Mathur, M.B., Vanderweele, T.J., 2019. Sensitivity Analysis for Publication Bias in Meta-Analyses.

Miller, C.M., 2011. Cash Transfers and Economic Growth: A Mixed Methods Analysis of Transfer Recipients and Business Owners in Malawi. Poverty and Public Policy 3.

Miller, C.M., Tsoka, M., Reichert, K., 2011. The Impact of the Social Cash Transfer Scheme on Food Security in Malawi. Food Policy 36, 230–238.

Mills, E.J., Adhvaryu, A., Jakiela, P., Birungi, J., Okoboi, S., Chimulwa, T.N.W., Wanganisi, J., Achilla, T., Popoff, E., Golchi, S., Karlan, D., 2018. Unconditional cash transfers for clinical and economic outcomes among HIV-affected Ugandan households. AIDS 32, 2023–2031.

Mostert, C.M., Vall Castello, J., 2020. Long Run Educational and Spillover Effects of Unconditional Cash Transfers: Evidence from South Africa. Econ. Hum. Biol. 36.

Nalwanga, R, Lund, R, Nalwanga, Rebecca, Lund, Ragnhild, 2018. Examining the impacts of grants on senior citizen beneficiaries in Kiboga District, Uganda. Dev. Pract. Pract. 28, 775–784.

Natali, L., Handa, S., Peterman, A., Seidenfeld, D., Tembo, G., 2018. Does money buy happiness? Evidence from an unconditional cash transfer in Zambia. SSM - Popul. Heal. 4, 225–235.

Nayab, D., Farooq, S., 2014. Effectiveness of Cash Transfer Programmes for Household Welfare in Pakistan: The Case of the Benazir Income Support Programme. Pak. Dev. Rev. 53, 145–174.

Nye, E., 2019. Meta-analysis lecture & tutorial.

Ohrnberger, J., Fichera, E., Sutton, M., Anselmi, L., 2020. The effect of cash transfers on mental health - new evidence from South Africa. BMC Public Health 20. https://doi.org/10.1186/s12889-020-08596-7

Okeke, E.N., Abubakar, I.S., 2020. Healthcare at the Beginning of Life and Child Survival: Evidence from a Cash Transfer Experiment in Nigeria. J. Dev. Econ. 143. https://doi.org/10.1016/j.jdeveco.2019.102426

Ozer, E., Fernald, L., Manley, J.G., Gertler, P.J., 2009. Effects of a conditional cash transfer program on children’s behavior problems. Pediatrics 123, e630–e637.

Ozer, E., Fernald, L., Weber, A., Flynn, E.P., Van der Weele, T.J., 2011. Does alleviating poverty affect mothers’ depressive symptoms? A quasi-experimental investigation of Mexico’s Oportunidades programme. Int. J. Epidemiol. 40, 1565–1576.

Özler, B., Hallman, K., Guimond, M.F., Kelvin, E.A., Rogers, M., Karnley, E., 2020. Girl Empower – A gender transformative mentoring and cash transfer intervention to promote adolescent wellbeing: Impact findings from a cluster-randomized controlled trial in Liberia. SSM - Popul. Heal. 10. https://doi.org/10.1016/j.ssmph.2019.100527

Pace, N., Daidone, S., Davis, B., Pellerano, L., 2019. Shaping Cash Transfer Impacts through “Soft-Conditions”: Evidence from Lesotho. J. Afr. Econ. 28, 39–69. https://doi.org/10.1093/jae/ejy009

Pais, P.S.M., Silva, F. de F., Teixeira, E.C., 2017. The Influence of Bolsa Familia Conditional Cash Transfer Program on Child Labor in Brazil. Int. J. Soc. Econ. 44, 206–221.

Pak, T.-Y., 2020. Social protection for happiness? The impact of social pension reform on subjective well-being of the Korean elderly. J. Policy Model. 42, 349–366.

Patel, L, Knijn, T, Van Wel, F, Patel, Leila, Knijn, Trudie, Van Wel, Frits, 2015. Child Support Grants in South Africa: A Pathway to Women’s Empowerment and Child Well-being? J. Soc. Policy 44, 377–397.

Paxson, C., Schady, N., 2010. Does Money Matter? The Effects of Cash Transfers on Child Development in Rural Ecuador. Econ. Dev. Cult. Change 59, 187–229.

Perova, E., Vakis, R., 2012. Five Years in Juntos: New Evidence on the Program’s Short and Long-Term Impacts. Econ. (Pontifical Cathol. Univ. Peru) 35, 53–82.

Pettifor, A., MacPhail, C., Hughes, J.P., Selin, A., Wang, J., F.X., G.-O., S.H., E., R.G., W., W., M., N., K., C., S., I., M., R., T., P., A., E., T., O., L., Y., A., S., T., K., K., 2016. The effect of a conditional cash transfer on HIV incidence in young women in rural South Africa (HPTN 068): a phase 3, randomised controlled trial. Lancet Glob. Heal. 4, e978–e988.

Pi Alperin, M.N., 2009. The Impact of Argentina’s Social Assistance Program Plan Jefes y Jefas de Hogar on Structural Poverty. Estud. Econ. 49–81.

Plagerson, S., Patel, V., Harpham, T., Kielmann, K., Mathee, A., 2011. Does money matter for mental health? Evidence from the Child Support Grants in Johannesburg, South Africa. Glob. Public Health 6, 760–776. https://doi.org/http://dx.doi.org/10.1080/17441692.2010.516267

Ponce, J., Bedi, A.S., 2010. The Impact of a Cash Transfer Program on Cognitive Achievement: The Bono de Desarrollo Humano of Ecuador. Econ. Educ. Rev. 29, 116–125.

Powell-Jackson, T., Pereira, S.K., Dutt, V., Tougher, S., Haldar, K., Kumar, P., 2016. Cash transfers, maternal depression and emotional well-being: quasi-experimental evidence from India’s Janani Suraksha Yojana programme. Soc. Sci. Med. 162, 210–218.

Rahman, M.M., 2014. Estimating the average treatment effect of social safety net programmes in Bangladesh. J. Dev. Stud. 50, 1550–1569.

Reis, M., 2010. Cash Transfer Programs and Child Health in Brazil. Econ. Lett. 108, 22–25. https://doi.org/10.1016/j.econlet.2010.04.009

Robertson, L., Mushati, P., Eaton, J.W., Dumba, L., Mavise, G., Makoni, J., Schumacher, C., Crea, T., Monasch, R., Sherr, L., Garnett, G.P., Nyamukapa, C., Gregson, S., 2013. Effects of unconditional and conditional cash transfers on child health and development in Zimbabwe: A cluster-randomised trial. Lancet 381, 1283–1292. https://doi.org/10.1016/S0140-6736(12)62168-0

Robertson, L., Mushati, P., Eaton, J.W., Dumba, L., Mavise, G., Makoni, J.C., Schumacher, C., Crea, T., Monasch, R., Sherr, L., Garnett, G.P., Nyamukapa, C., Gregson, S., 2012. Conditional cash transfers improve birth registration and school attendance amongst orphans and vulnerable children in Manicaland, Zimbabwe. J. Int. AIDS Soc., ["19th International AIDS Conference. Washington, DC United States.", “(var.pagings).”] 15, 158–159.

Robinson, J., 2012. Limited insurance within the household: Evidence from a field experiment in Kenya. Am. Econ. J. Appl. Econ. 4, 140–164. https://doi.org/10.1257/app.4.4.140

Rocha, C., Montoya, R., Zevallos, K., Curatola, A., Ynga, W., Franco, J., Fernandez, F., Becerra, N., Sabaduche, M., Tovar, M.A., Ramos, E., Tapley, A., Allen, N., Onifade, D.A., Acosta, C.D., Maritz, M., Concha, D.F., Schumacher, S.G., Evans, C.A., 2011. The Innovative Socio-economic Interventions Against Tuberculosis (ISIAT) project: An operational assessment. Int. J. Tuberc. Lung Dis. 15, S50–S57. https://doi.org/10.5588/ijtld.10.0447

Roy, S., Hidrobo, M., Hoddinott, J.F., Ahmed, A., 2017. Transfers, behavior change communication, and intimate partner violence: postprogram evidence from rural Bangladesh. (No. 01676), IFPRI - Discussion Papers. Washington, D.C.

Salehi-Isfahani, D., Mostafavi-Dehzooei, M.H., 2018. Cash Transfers and Labor Supply: Evidence from a Large-Scale Program in Iran. J. Dev. Econ. 135, 349–367.

Salinas-Rodríguez, A., Torres-Pereda, M.D.P., Manrique-Espinoza, B., Moreno-Tamayo, K., Solís, M.M.T.R., 2014. Impact of the non-contributory social pension program 70 y más on older adults’ mental well-being. PLoS One 9, e113085. https://doi.org/10.1371/journal.pone.0113085

Schatz, E., Gomez-Olive, X., Ralston, M., Menken, J., Tollman, S., 2012. The impact of pensions on health and wellbeing in rural South Africa: Does gender matter? Soc. Sci. Med. 75, 1864–1873.

Sedlmayr, R., Shah, A., Sulaiman, M., 2020. Cash-plus: Poverty impacts of alternative transfer-based approaches. J. Dev. Econ. 144. https://doi.org/10.1016/j.jdeveco.2019.102418

Shangani, S., Operario, D., Genberg, B., Kirwa, K., Midoun, M., Atwoli, L., Ayuku, D., Galarraga, O., Braitstein, P., 2017. Unconditional government cash transfers in support of orphaned and vulnerable adolescents in western Kenya: is there an association with psychological wellbeing? PLoS One 12, e0178076. https://doi.org/http://dx.doi.org/10.1371/journal.pone.0178076

Singh, N., 2019. Gender, Intra-household Discrimination and Cash Transfer Schemes: The Case of Indian Punjab. Economies 7, 1–13.

Sugiyama, N.B., Hunter, W., 2020. Do Conditional Cash Transfers Empower Women? Insights from Brazil’s Bolsa Família. Lat. Am. Polit. Soc. 62, 53–74.

Sulaiman, M., Goldberg, N., Karlan, D., de Montesquiou, A., 2016. Eliminating Extreme Poverty: Comparing the Cost-Effectiveness of Livelihood, Cash Transfer, and Graduation Approaches (No. 11), CGAP Report.

Szulc, A., 2012. Social Policy and Poverty: Checking the Efficiency of the Social Assistance System in Poland. East. Europ. Econ. 50, 66–92.

Tagliati, F., 2019. Child labor under cash and in-kind transfers: evidence from rural Mexico, Banco de España Working Papers, 1935. Working Papers Homepage, Working Papers: 1935, Banco de Espana.

Taylor, J.E., Thome, K., Filipski, M., 2016. Local Economy-Wide Impact Evaluation of Social Cash Transfer Programmes, in: Yablonski, J., Handa, S., Winters, P., Winder Rossi, N., Davis, B., Hypher, N. (Eds.), From Evidence to Action: The Story of Cash Transfers and Impact Evaluation in Sub-Saharan Africa. FAO, Rome, pp. 94–114.

Thompson, H., 2014. Cash for protection: Cash transfer programs can promote child protection outcomes. Child Abus. Negl. 38, 360–371. https://doi.org/10.1016/j.chiabu.2014.01.013

Tiwari, S., 2019. Long-Term Effects of Temporary Income Shocks on Food Consumption and Subjective Well-Being. J. Dev. Stud. 55, 1687–1707.

Tsaneva, M., Balakrishnan, U., 2019. The Effect of a Workfare Programme on Psychological Wellbeing in India. J. Dev. Stud. 55, 2593–2609.

Undurraga, E.A., Behrman, J.R., Leonard, W.R., Godoy, R.A., 2016. The effects of community income inequality on health: evidence from a randomized control trial in the Bolivian Amazon. Soc. Sci. Med. 149, 66–75.

Unnikishnan, V., Imai, K.S., 2018. Does the Old Age Pension Scheme Improve Household Welfare? Evidence from India (No. DP2018-20), Research Institute for Economics & Business Administration Discussion Paper Series.

Urrea, M.A., Maldonado, J.H., 2011. Vulnerability and Risk Management: The Importance of Financial Inclusion for Beneficiaries of Conditional Transfers in Colombia. Can. J. Dev. Stud. 32, 381–398.

Valadez-Martinez, L.J., 2016. Household Income Trajectories, PROGRESA-Oportunidades, and Child Well-Being at Pre-school Age in Rural Mexico. J. Hum. Dev. Capab. 17, 516–539. https://doi.org/10.1080/19452829.2016.1225701

Viechtbauer, W., 2010. Conducting meta-analyses in R with the metafor package. J. Stat. Softw. 36, 1–48. https://doi.org/10.18637/jss.v036.i03

Waqas, M., Awan, M.S., 2018. Access to better health? The impact of the Benazir Income Support Programme in Pakistan. Asio Pacific J. Public Adm. 40, 74–81.

Whetten, J., Fontenla, M., Villa, K., 2019. Opportunities for Higher Education: The Ten-Year Effects of Conditional Cash Transfers on Upper-Secondary and Tertiary Enrollments. Oxford Dev. Stud. 47, 222–237.

Yang, L., 2013. The Urban Dibao Programme in China: Targeting and Its Effect. Indian J. Labour Econ. 56, 597–615.

Zhang, Z, Luo, Y, Robinson, D, Zhang, Zhaohua, Luo, Yuxi, Robinson, Derrick, 2019. Who Are the Beneficiaries of China’s New Rural Pension Scheme? Sons, Daughters, or Parents? Int. J. Environ. Res. Public Health 16, 1–16.
