# Supplementary material for: Do cash transfers alleviate common mental disorders in low- and middle-income countries? A systematic review and meta-analysis
Source: PLoS One. 2023 Feb 22;18(2):e0281283. doi: 10.1371/journal.pone.0281283 (PMC9946251; doi:10.1371/journal.pone.0281283)
Supplement: S3 File — (PDF) [file pone.0281283.s003.pdf]

Systematic review

1. * Review title.

Give the title of the review in English

Do income transfers alleviate mental health problems? A systematic review and meta-analysis of cash transfer programs in low- and middle-income countries

2. Original language title.

For reviews in languages other than English, give the title in the original language. This will be displayed with the English language title.

3. * Anticipated or actual start date.

Give the date the systematic review started or is expected to start.

03/03/2020

4. * Anticipated completion date.

Give the date by which the review is expected to be completed.

30/12/2020

5. * Stage of review at time of this submission.

Tick the boxes to show which review tasks have been started and which have been completed. Update this field each time any amendments are made to a published record.

Reviews that have started data extraction (at the time of initial submission) are not eligible for inclusion in PROSPERO. If there is later evidence that incorrect status and/or completion date has been supplied, the published PROSPERO record will be marked as retracted.

This field uses answers to initial screening questions. It cannot be edited until after registration.

The review has not yet started: No

Review stage	Started	Completed
Preliminary searches	Yes	Yes
Piloting of the study selection process	Yes	Yes
Formal screening of search results against eligibility criteria	Yes	No
Data extraction	No	No
Risk of bias (quality) assessment	Yes	No
Data analysis	No	No

Provide any other relevant information about the stage of the review here.

6. * Named contact.

The named contact is the guarantor for the accuracy of the information in the register record. This may be any member of the review team.

Clara Wollburg

Email salutation (e.g. "Dr Smith" or "Joanne") for correspondence:

Ms Wollburg

7. * Named contact email.

Give the electronic email address of the named contact.

clara.wollburg@gmail.com

8. Named contact address

Give the full institutional/organisational postal address for the named contact.

Wolfson College, Linton Road, OX2 6UD, Oxford

9. Named contact phone number.

Give the telephone number for the named contact, including international dialling code.

00491727471991

10. * Organisational affiliation of the review.

Full title of the organisational affiliations for this review and website address if available. This field may be completed as 'None' if the review is not affiliated to any organisation.

University of Oxford

Organisation web address:

11. * Review team members and their organisational affiliations.

Give the personal details and the organisational affiliations of each member of the review team. Affiliation refers to groups or organisations to which review team members belong. **NOTE: email and country now MUST be entered for each person, unless you are amending a published record.**

Ms Clara Wollburg. University of Oxford
Professor Janina Steinert. Technische Universität München

12. * Funding sources/sponsors.

Details of the individuals, organizations, groups, companies or other legal entities who have funded or sponsored the review.

None

Grant number(s)

State the funder, grant or award number and the date of award

13. * Conflicts of interest.

List actual or perceived conflicts of interest (financial or academic).

None

14. Collaborators.

Give the name and affiliation of any individuals or organisations who are working on the review but who are not listed as review team members. **NOTE: email and country must be completed for each person, unless you are amending a published record.**

15. * Review question.

State the review question(s) clearly and precisely. It may be appropriate to break very broad questions down into a series of related more specific questions. Questions may be framed or refined using PI(E)COS or similar where relevant.

Can cash transfer programs (CTPs) improve mental health outcomes for low-income recipients in low- and middle-income countries (LMICs) compared to inactive control groups?

- Population & Context: Low-income households or individuals receiving CTPs in LMICs as defined by the World Bank

- Intervention: Unconditional and conditional CTPs in LMICs

- Comparison: Inactive control group receiving treatment-as-usual, no treatment, or treatment at a later stage (wait-list control)

- Outcomes: Mental health outcomes post-intervention, measured by validated instruments: Depression, anxiety, and stress. While I focus on post-intervention outcomes, I will also incorporate follow-up measurements if available, as they can provide important insights into longer-term effects.

16. * Searches.

State the sources that will be searched (e.g. Medline). Give the search dates, and any restrictions (e.g. language or publication date). Do NOT enter the full search strategy (it may be provided as a link or attachment below.)

I will search the following electronic databases using the syntax provided in table 3, adjusted to each

database: Scopus, Web of Science, MEDLINE, Embase, PsycINFO, Global Health, Econlit, and Ideas/Repec. I will also search the following websites and databases to capture grey literature: The Transfer Project, the Abdul Latif Jameel Poverty Action Lab (J-PAL), Innovations for Poverty Action (IPA), the 3ie Repository of Impact Evaluations, which comprise impact evaluation studies in LMICs. Finally, I will search the bibliography of included studies to identify further relevant articles.

17. URL to search strategy.

Upload a file with your search strategy, or an example of a search strategy for a specific database, (including the keywords) in pdf or word format. In doing so you are consenting to the file being made publicly accessible. Or provide a URL or link to the strategy. Do NOT provide links to your search **results**.

https://www.crd.york.ac.uk/PROSPEROFILES/186955_STRATEGY_20200518.pdf

Alternatively, upload your search strategy to CRD in pdf format. Please note that by doing so you are consenting to the file being made publicly accessible.

Do not make this file publicly available until the review is complete

18. * Condition or domain being studied.

Give a short description of the disease, condition or healthcare domain being studied in your systematic review.

Mental ill-health is characterized as one of the major non-communicable diseases in low- and middle-income countries (LMICs), with its relative burden of disease estimated to grow significantly in the next decade (Lund et al., 2018; Plagerson, Patel, Harpham, Kielmann, & Mathee, 2011). Within LMICs, poorer populations are especially vulnerable to mental health problems due to their exposure to stressors such as food insecurity, social exclusion, financial strain, and violence (Patel et al., 2009). At the same time, low-income households may have less resources available to protect themselves from and cope with such stressors (Hjelm, Handa, Hoop, & Palermo, 2017). Accordingly, the prevalence of common mental disorders, such as depression and anxiety, is disproportionality high among poor populations (Lund et al., 2018; Patel et al., 2009). Moreover, low-income household exhibit higher levels of stress hormones, such as cortisol, and report lower quality of life and psychosocial wellbeing (Haushofer & Fehr, 2014).

19. * Participants/population.

Specify the participants or populations being studied in the review. The preferred format includes details of both inclusion and exclusion criteria.

I focus on low-income or vulnerable recipients of CTPs living in LMICs. I include both adults and adolescents, who are CTP recipients. I do not include children under the age of 12, but I will review CTPs aimed at increasing school attendance, since those studies may test for caregivers' mental health.

20. * Intervention(s), exposure(s).

Give full and clear descriptions or definitions of the interventions or the exposures to be reviewed. The preferred format includes details of both inclusion and exclusion criteria.

I will examine CTPs targeted at low-income and vulnerable households or individuals in LMICs including social protection programs, such as social pensions and child support grants. I include both unconditional CTPs, i.e. no conditions have to be met to receive the financial support, and conditional CTPs, i.e. conditions are attached to the transfers regarding e.g. school attendance or vaccination of children (Khan, Hazra, Kant, & Ali, 2016). I will not examine “cash plus” programs, which combine CTPs with complementary interventions such as counselling (Roelen et al., 2017), as it is difficult to disentangle the causal effects of cash transfers on mental health.

21. * Comparator(s)/control.

Where relevant, give details of the alternatives against which the intervention/exposure will be compared (e.g. another intervention or a non-exposed control group). The preferred format includes details of both inclusion and exclusion criteria.

Participants in inactive control groups, who do not receive the CTP, serve as comparison group for the review. They could be receiving either treatment-as-usual, i.e. access to standard care and social services, or treatment at a later stage of the trial (waitlist control). I do not include active control groups receiving alternative interventions, e.g. medication, which are likely to impact mental health outcomes, as this makes causal inference about the effects of CTPs difficult.

22. * Types of study to be included.

Give details of the study designs (e.g. RCT) that are eligible for inclusion in the review. The preferred format includes both inclusion and exclusion criteria. If there are no restrictions on the types of study, this should be stated.

I will include randomized controlled trials (RCTs) and Cluster-RCTs. Depending on the number of identified studies, I will decide whether to constrain eligibility to RCTs / Cluster-RCTs or to extend the scope to experimental and quasi-experimental studies with credible comparison groups, e.g. regression-discontinuity, difference-in-difference, and matching designs (Bastagli et al., 2016). I choose these study types because they offer a comparable counterfactual and, thus, tend to allow for robust causal assumptions about the effectiveness of CTPs (Bastagli et al., 2016). Should I include quasi-experimental studies, I will conduct a sensitivity analyses without quasi-experimental studies. I will not include qualitative, descriptive, or theoretical studies.

23. Context.

Give summary details of the setting or other relevant characteristics, which help define the inclusion or exclusion criteria.

24. * Main outcome(s).

Give the pre-specified main (most important) outcomes of the review, including details of how the outcome is defined and measured and when these measurement are made, if these are part of the review inclusion criteria.

I will include studies examining the following mental health outcomes post-intervention: (1) stress, measured

by validated surveys, e.g. Cohen's perceived stress scale and (2) depression and anxiety measured by validated surveys, e.g. General Health Questionnaire-12 or CES-D. In case only aggregated mental health measures will be available, I will include these in a separate meta-analyses. While I focus on post-intervention outcomes, I will incorporate follow-up measurements in narrative synthesis, as they can provide important insights into longer-term effects. Other mental health outcomes reported in included studies, e.g. subjective wellbeing, will be included as part of the narrative synthesis.

* Measures of effect

Please specify the effect measure(s) for you main outcome(s) e.g. relative risks, odds ratios, risk difference, and/or 'number needed to treat.

Odds ratios, regression coefficients, mean differences

25. * Additional outcome(s).

List the pre-specified additional outcomes of the review, with a similar level of detail to that required for main outcomes. Where there are no additional outcomes please state 'None' or 'Not applicable' as appropriate to the review

Not applicable

* Measures of effect

Please specify the effect measure(s) for you additional outcome(s) e.g. relative risks, odds ratios, risk difference, and/or 'number needed to treat.

Not applicable

26. * Data extraction (selection and coding).

Describe how studies will be selected for inclusion. State what data will be extracted or obtained. State how this will be done and recorded.

Using the online tool Rayyan, I will screen abstracts and titles of all retrieved records (Ouzzani, Hammady, Fedorowicz, & Elmagarmid, 2016). A full-text screening of the remaining articles will be conducted and a list with excluded studies and reasons for exclusion will be provided. A second reviewer will screen 10% of titles and abstracts, which will be randomly selected, in order to reduce bias in the study selection process. Disagreements will be resolved through discussion. For all included studies, data will be extracted using the Effective Practice and Organisation of Care (EPOC) extraction form (EPOC, 2002).

27. * Risk of bias (quality) assessment.

State which characteristics of the studies will be assessed and/or any formal risk of bias/quality assessment tools that will be used.

I will assess the quality of included RCTs using the Cochrane Risk of Bias Tool version 2 (RoB 2) for randomized studies (Sterne et al., 2019). The RoB 2 evaluates the following domains: (1) bias arising from the randomization process; (2) bias due to deviations from intended interventions; (3) bias due to missing outcome data; (4) bias in measurement of the outcome; (5) bias in selection of the reported result. Cluster-RCTs and quasi-experimental studies will be assessed with the adapted tools RoB 2 for cluster-randomized

trials and Risk Of Bias In Non-randomized Studies of Interventions (ROBINS-I) (Sterne, Hernán, McAleenan, Reeves, & Higgins, 2019). A second reviewer will conduct a ROB assessment of a random 10% of included studies, in order to reduce bias. Based on the RoB assessment, meta-analysis will be performed with and without studies of high risk of bias, to test whether the overall results change when lower quality studies are excluded (Petticrew & Roberts, 2008). Should I include more than 10 studies in the meta-analysis, I will draw contour-enhanced funnel plots, which map effect sizes against standard errors of studies, in order to test for reporting bias (Higgins & Green, 2019).

28. * Strategy for data synthesis.

Describe the methods you plan to use to synthesise data. This **must not be generic text** but should be **specific to your review** and describe how the proposed approach will be applied to your data. If meta-analysis is planned, describe the models to be used, methods to explore statistical heterogeneity, and software package to be used.

In order to produce a quantitative summary of included studies, I will conduct a random-effects meta-analysis in R, using the metafor package. Since I include studies from different countries, settings, and target groups, the random-effects model is a better estimation for my analysis as it does not assume that all studies represent variations of the same intervention effect (Higgins & Green, 2019). In case of data dependency issues, which occur for example when studies measure several mental health outcomes within the same sample, I will use a multilevel meta-analysis (MMA) or Robust Variance Estimation (RVE). MMA and RVE have the advantage of accounting for dependent effect sizes and, thus, producing more accurate estimations of the overall effect (Moeyaert et al., 2017). I will convert or, where necessary, calculate effect sizes of eligible studies into a common standardized effect size measure, Cohen’s d, using Wilson’s effect size calculator (Wilson, 2014). Weighted means with 95%-confidence intervals will be reported (Higgins & Green, 2019). I assess heterogeneity, i.e. the variability across studies, using the I^2 and $?^2$ statistic. $?^2$ measures the between-study variability in random effects models; I^2 estimates the percentage of between-study variability that is a result of heterogeneity rather than random error (Higgins & Green, 2019).

29. * Analysis of subgroups or subsets.

State any planned investigation of ‘subgroups’. Be clear and specific about which type of study or participant will be included in each group or covariate investigated. State the planned analytic approach.

If the number of included studies allows (k 10), I will further perform a random-effects meta-regression to compare the relative effectiveness of conditional and unconditional CTPs.

30. * Type and method of review.

Select the type of review, review method and health area from the lists below.

Type of review

Cost effectiveness
No

Diagnostic

No

Epidemiologic
No

Individual patient data (IPD) meta-analysis
No

Intervention
Yes

Meta-analysis
Yes

Methodology
No

Narrative synthesis
Yes

Network meta-analysis
No

Pre-clinical
No

Prevention
No

Prognostic
No

Prospective meta-analysis (PMA)
No

Review of reviews
No

Service delivery
No

Synthesis of qualitative studies
No

Systematic review
Yes

Other
No

Health area of the review

Alcohol/substance misuse/abuse
No

Blood and immune system
No

Cancer
No

Cardiovascular
No

Care of the elderly
No

Child health
No

Complementary therapies
No

COVID-19
No

Crime and justice
No

Dental
No

Digestive system
No

Ear, nose and throat
No

Education
No

Endocrine and metabolic disorders
No

Eye disorders
No

General interest
No

Genetics
No

Health inequalities/health equity
No

Infections and infestations
No

International development
No

Mental health and behavioural conditions
Yes

Musculoskeletal
No

Neurological
No

Nursing
No

Obstetrics and gynaecology
No

Oral health

No

Palliative care
No

Perioperative care
No

Physiotherapy
No

Pregnancy and childbirth
No

Public health (including social determinants of health)
No

Rehabilitation
No

Respiratory disorders
No

Service delivery
No

Skin disorders
No

Social care
No

Surgery
No

Tropical Medicine
No

Urological
No

Wounds, injuries and accidents
No

Violence and abuse
No

31. Language.

Select each language individually to add it to the list below, use the bin icon to remove any added in error.
English

There is not an English language summary

32. * Country.

Select the country in which the review is being carried out. For multi-national collaborations select all the countries involved.

England
Germany

33. Other registration details.

Name any other organisation where the systematic review title or protocol is registered (e.g. Campbell, or The Joanna Briggs Institute) together with any unique identification number assigned by them. If extracted data will be stored and made available through a repository such as the Systematic Review Data Repository (SRDR), details and a link should be included here. If none, leave blank.

34. Reference and/or URL for published protocol.

If the protocol for this review is published provide details (authors, title and journal details, preferably in Vancouver format)

Add web link to the published protocol.

Or, upload your published protocol here in pdf format. Note that the upload will be publicly accessible.

No I do not make this file publicly available until the review is complete

Please note that the information required in the PROSPERO registration form must be completed in full even if access to a protocol is given.

35. Dissemination plans.

Do you intend to publish the review on completion?

Yes

Give brief details of plans for communicating review findings.?

36. Keywords.

Give words or phrases that best describe the review. Separate keywords with a semicolon or new line. Keywords help PROSPERO users find your review (keywords do not appear in the public record but are included in searches). Be as specific and precise as possible. Avoid acronyms and abbreviations unless these are in wide use.

Cash transfers; Mental health; Depression; Anxiety; Stress; Poverty; Common Mental Disorders

37. Details of any existing review of the same topic by the same authors.

If you are registering an update of an existing review give details of the earlier versions and include a full bibliographic reference, if available.

38. * Current review status.

Update review status when the review is completed and when it is published.New registrations must be ongoing so this field is not editable for initial submission.
Please provide anticipated publication date

Review_Ongoing

39. Any additional information.

Provide any other information relevant to the registration of this review.

40. Details of final report/publication(s) or preprints if available.

Leave empty until publication details are available OR you have a link to a preprint (NOTE: this field is not editable for initial submission). List authors, title and journal details preferably in Vancouver format.

Give the link to the published review or preprint.
