# Supplementary material for: Do cash transfers alleviate common mental disorders in low- and middle-income countries? A systematic review and meta-analysis
Source: PLoS One. 2023 Feb 22;18(2):e0281283. doi: 10.1371/journal.pone.0281283 (PMC9946251; doi:10.1371/journal.pone.0281283)
Supplement: Attachment — Submitted filename: PLOS1_Response Letter_2.docx [file pone.0281283.s005.docx]

**Response Letter**

**Do cash transfers alleviate common mental disorders in low- and middle-income countries? A systematic review and meta-analysis**

*PLOS ONE - PONE-D-21-00721R1*

Dear Reviewer, dear Editors,

Thank you again for your valuable feedback and for pointing us to the recent systematic review by Zimmerman et al. on the mental health effects of cash transfers in young people and children. We have incorporated the review as suggested and have added the following paragraph to the introduction:

“Zimmerman et al. (2021) published a systematic review and meta-analysis on the effects of cash transfers on mental health outcomes of children and young people (35). Since Zimmerman and colleagues focus on children and adolescents, however, their analysis does not include many of the studies analyzed in this review.” (Section 1, lines 108 – 111, revised manuscript with track changes)

Moreover, we have included the findings in the discussion section:

“Can cash transfers play a role in alleviating common mental disorders in LMICs? This systematic review summarized the evidence on the effects of CTPs on stress, anxiety, and depressive symptoms in adults and adolescents who live in poverty. Meta-analysis of 11 studies and pooling data from 22,488 participants shows that cash grants significantly improve depression and anxiety post-intervention. Following GRADE-criteria, the overall confidence is judged to be moderate, due to some indication of publication bias and since meta-analyzed trials carried intermediate to high risk of bias. The magnitude of the effect is comparable to meta-analysis on other distal outcomes in the cash transfer literature, e.g. on child labor (91) and test scores (29). Moreover, our effect sizes are similar to those found by Ridley et al. (12) and McGuire et al. (30), who also report significant positive impacts on common mental disorders. Zimmerman et al. (35), on the other hand, find positive, but much smaller and insignificant aggregate effects on depressive symptoms in children and young people. The authors note, however, that the validity of the aggregate effect size is limited due to substantial heterogeneity.” (Section 4.1, lines 579 – 591, revised manuscript with track changes)

Reviewer 2 also noted the following: “*A minor observation is that it would have been extremely helpful to compare the effect sizes with the one of (universal) mental health intervention, and make the paper even more relevant.”*

Many thanks for this suggestion. In our original submission, we did compare effect sizes of our review to reviews looking at the effectiveness of psychological therapies and antidepressants in LMICs (Moncrieff, Wessely, & Hardy, 2004; Purgato et al., 2018). However, reviewer 1 rightly pointed us to the limited comparability of samples from CTP studies and samples from mentally-ill populations in LMICs: *“cash transfers are not targeted to mentally ill populations and provide an average treatment effect whereas treatment with anti-depressants are targeted towards a sub-sample. Effects will be larger for those in need which are at the end of the effect distribution. To illustrate, a recent analysis which moved beyond the mean found a four-times increased of a positive cash-transfer programme effect on mental health for those with worst mental health conditions compared to the mean-effect (Ohrnberger et al. 2020). –* [*https://doi.org/10.1093/heapol/czaa079*](https://doi.org/10.1093/heapol/czaa079)*)”*

In response to this comment, we decided not to compare effect sizes to those of mental health interventions.

**References:**

Baird, S., Ferreira, F. H. G., Özler, B., & Woolcock, M. (2013). Relative Effectiveness of Conditional and Unconditional Cash Transfers for Schooling Outcomes in Developing Countries: A Systematic Review. *Campbell Systematic Reviews*, *9*(1), 1–124. https://doi.org/10.4073/csr.2013.8

Kabeer, N., & Waddington, H. (2015). Economic impacts of conditional cash transfer programmes: a systematic review and meta-analysis. *Journal of Development Effectiveness*, *7*(3), 290–303. https://doi.org/10.1080/19439342.2015.1068833

McGuire, J., Kaiser, C., & Bach-Mortensen, A. (2020). *The impact of cash transfers on subjective well-being and mental health in low- and middle- income countries: A systematic review and meta-analysis*. Retrieved from https://osf.io/preprints/socarxiv/ydr54/

Moncrieff, J., Wessely, S., & Hardy, R. (2004). Active placebos versus antidepressants for depression. *Cochrane Database of Systematic Reviews*, *1*(CD003012). https://doi.org/10.1002/14651858.CD003012.pub2

Purgato, M., Gastaldon, C., Papola, D., van Ommeren, M., Barbui, C., & Tol, W. A. (2018). Psychological therapies for the treatment of mental disorders in low‐ and middle‐income countries affected by humanitarian crises. *Cochrane Database of Systematic Reviews*, *7*(CD011849). https://doi.org/10.1002/14651858.CD011849.pub2

Ridley, M., Rao, G., Schilbach, F., & Patel, V. (2020). Poverty, depression, and anxiety: Causal evidence and mechanisms. *Science*, *370*(6522), eaay0214. https://doi.org/10.1126/science.aay0214

Zimmerman, A., Garman, E., Avendano-Pabon, M., Araya, R., Evans-Lacko, S., McDaid, D., … Lund, C. (2021). The impact of cash transfers on mental health in children and young people in low-income and middle-income countries: A systematic review and meta-analysis. *BMJ Global Health*, *6*(4), 1–16. https://doi.org/10.1136/bmjgh-2020-004661
